# Effects of using medication reminder technologies by home-dwelling older citizens: a systematic review

**DOI:** 10.1093/ageing/afag007

**Published:** 2026-02-05

**Authors:** Olli Salmensuu, Jenni Isotalo, Mieke Rijken, Virva Hyttinen-Huotari, Minna Kaarakainen, Ismo Linnosmaa

**Affiliations:** Department of Health and Social Management, University of Eastern Finland Faculty of Social Sciences and Business Studies, Kuopio, Finland; Department of Health and Social Management, University of Eastern Finland Faculty of Social Sciences and Business Studies, Kuopio, Finland; Department of Health and Social Management, University of Eastern Finland Faculty of Social Sciences and Business Studies, Kuopio, Finland; NIVEL, Utrecht, The Netherlands; Department of Health and Social Management, University of Eastern Finland Faculty of Social Sciences and Business Studies, Kuopio, Finland; Department of Health and Social Management, University of Eastern Finland Faculty of Social Sciences and Business Studies, Kuopio, Finland; THL, Helsinki, Finland; Department of Health and Social Management, University of Eastern Finland Faculty of Social Sciences and Business Studies, Kuopio, Finland; THL, Helsinki, Finland

**Keywords:** medication reminder technologies, older citizens, user experiences, health outcomes, health service use, costs, systematic review, older people

## Abstract

**Objective:**

Population ageing has increased the need for solutions that support independent living, with medication management being a major challenge. We assessed the effects of reminder technologies among home-dwelling older citizens on outcomes within the Quintuple Aim domains: user experiences, care professional experiences, health/wellbeing, health and social service utilisation/costs and equity.

**Methods:**

We searched databases (Scopus, CENTRAL, PubMed, Web of Science, CINAHL, PsycINFO and Cochrane Reviews) from 1.1.2017 to 29.9.2025. Two authors extracted relevant data and assessed the quality of the included studies. We assessed the evidence using a four-level quality rating scale: strong, moderate, limited or no evidence.

**Results:**

Twenty-three original studies and nine systematic reviews were included, resulting in 43 original studies. Significant beneficial effects on health outcomes were observed in 20 out of 40 studies, and on service utilisation in one out of four studies. Significant effects on patient/carer experiences and cost-effectiveness were not found, whereas no study assessed effects on professional experiences or equity. Only for clinical health outcomes, in particular systolic blood pressure and physical symptoms, the effectiveness of reminders reached moderate evidence.

**Conclusion:**

While clinical health benefits have been observed, more high-quality research is needed to determine whether medication reminder technologies can help more broadly to respond to the challenges of population ageing, including the high pressure on health services and related expenditures.

## Key Points

- Moderate evidence supports the use of medication reminders in improving systolic blood pressure and physical symptoms.
- Evidence of medication reminders impacting service use, cost-effectiveness and user or carer experiences—remains limited.
- More high-quality studies are needed to assess the real-world impact of medication reminders on ageing populations and health care systems.
- Policymakers should consider supporting the integration of low-cost medication reminder technologies into national ageing strategies.

## Introduction

### Background and rationale for the review

A great majority of older adults wish to live in their homes for as long as possible [1, 2], which increases the need for effective and cost efficient solutions to support independent living and prevent institutionalisation. The challenges in managing complex medication administration regimens [3, 4] make the use of technology a promising solution. Medication management technologies have shown promise in improving medication adherence [5–7], and may reduce healthcare expenditures through their positive health effects [8–10]. While adherence is important, the effects of medication management technologies on health and wellbeing, service use and costs need more clarity.

### Objective

Older adults are an important target group for reminder technologies, which are offering a low entry, yet high-technology, solution to personal medication management. This systematic review aims to provide insights in the effects of medication reminder technologies on outcomes within the five domains of the Quintuple Aim framework for health care improvement [11]: (i) Care experiences of service users and carers, e.g. satisfaction of older adults, burden of family/carers; (ii) Health and social service utilisation, costs and cost-effectiveness; (iii) Health and wellbeing; (iv) Wellbeing and experiences of care professionals (e.g. workload, job satisfaction); and (v) Equity.

### Scope of the review

This review focuses on reminder technologies that specifically support personal medication management, rather than being just one component of broader lifestyle interventions, because these broader interventions could mask the effects of the medication reminder functionality [12, 13]. We did not study the effects on medication adherence, as this has already been frequently studied, but focused on outcomes within all domains of the Quintuple Aim framework because of their relevance for service design and policymaking.

## Methods

Studies on the effectiveness of personal medication management devices often study either medication reminders or various dispensing approaches. The difficulty in searching the literature and synthesising the evidence of their effectiveness is that often the specific functionality of the device is not revealed in the title and abstract of studies, which carries the risk of falsely excluding relevant studies in the phase of title and abstract screening. Therefore, we screened the literature on medication reminders in parallel to our screening for another systematic review on medication dispensing technologies, in both the title/abstract screening and full-text screening. In this article, we describe the effects of reminder technologies, while the effects of dispenser technologies have been reported elsewhere [14].

### Search strategy and data sources

With the help of an information specialist, we selected and searched the following databases: PubMed, Cochrane (CENTRAL and Reviews), Scopus, WoS, CINAHL, PsycINFO. We limited the search to studies published from 2017 onward. This period saw the transition from simpler, often short message service (SMS)-based, interventions to the development, marketing and adoption of smartphone app-based medication management technologies [15]. The full search strategy can be found in the Appendix, following Table A4.

### Study selection

Inclusion and exclusion criteria were the following (Table 1).

**Table 1:** Inclusion and exclusion criteria.

Inclusion criteria	Exclusion criteria
(Participants) Study participants include adults aged ≥65 years living in their own home. (Interventions) Study focuses on, or a substantial part of the intervention consists of, using a medication reminder or alert. (Comparators) Study is an experimental or observational study including a comparative element (e.g. data collected from a control group, data collected prior to the implementation of the intervention, reference data) or the study is a systematic review that includes at least one original study passing all inclusion criteria.	(Outcomes) Study does not analyse or synthesise effects on outcomes within at least one of the Quintuple Aim domains: (i) Care experiences of older adults and caregivers; (ii) Wellbeing and experiences of care professionals (e.g. workload, job satisfaction); (iii) Health and/or wellbeing outcomes; (iv) Use of health and/or social care/costs/cost-effectiveness; (v) Equity.

**Figure 1:**
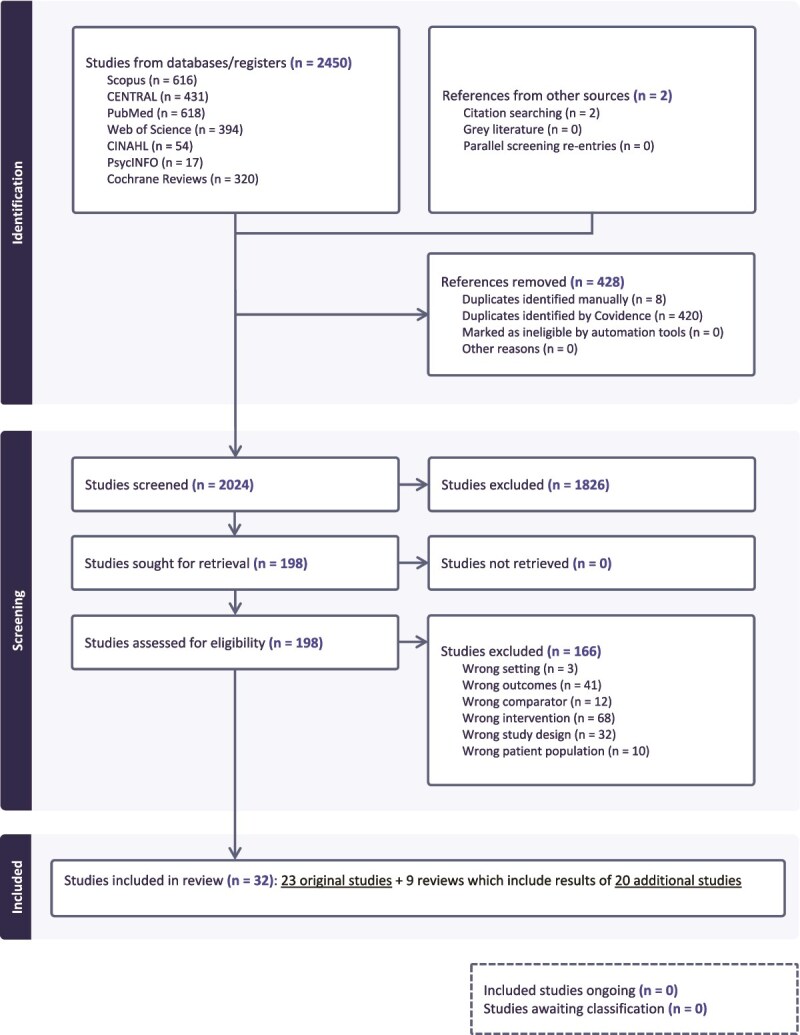
PRISMA flow diagram on study selection.

The searches initially identified 2450 records (see Appendix Table A1 for the completed Preferred Reporting Items for Systematic Reviews and Meta-analyses (PRISMA) checklist and Figure 1 for the PRISMA flow diagram on study selection). All retrieved records were exported into Covidence software and duplicates were removed. Both title/abstract and full text screening were conducted by two reviewers (OS and VHH/JI) independently against the predetermined inclusion and exclusion criteria. Ambiguities concerning the inclusion of articles were discussed by the two reviewers, and, where needed, with the wider research team to reach consensus. Full text screening of 198 articles resulted in 32 included studies, of which 23 described original studies and nine were systematic reviews identifying another 20 original studies that met our criteria. We extracted data from original studies included in the nine reviews to allow a more precise synthesis of their results as well as the use of their bias assessments. Appendix Table A2 lists the 166 studies that were excluded at this stage with reasons.

### Data extraction and quality assessment

Study results were extracted independently by two reviewers (OS and JI) and subsequently discussed to reach consensus. In addition, one reviewer (OS) extracted some general information. Two reviewers (OS and JI) also assessed the risk of bias, discussing with the research team any ambiguities. We assessed the quality of the systematic reviews using the AMSTAR2 tool for systematic reviews that cover both randomised and non-randomised studies of healthcare interventions [16], whereas for the initially identified 23 original studies we used the Mixed Methods Appraisal Tool (MMAT [17]).

### Synthesis of results and evidence levels

We synthesised the results narratively, as conducting meta-analyses was prevented by heterogeneity in populations, quality of randomised controlled trials (RCTs) and missing outcome information. We assessed the strength of the evidence using the following classification: 1. Strong evidence: Consistent beneficial effects in at least three statistically significant high-quality (score 4 or 5, or similar ranking) RCTs; 2. Moderate evidence: Consistent beneficial evidence in multiple studies, including at least one statistically significant high-quality RCT; 3. Limited evidence: Consistent beneficial findings including at least one statistically significant finding; 4. No evidence: Non-significant or inconsistent findings across studies. Consistency was defined as at least 75% of studies showing results in the same direction.

## Results

### Study and intervention characteristics

The 23 original studies are listed in Table 2 and the nine systematic reviews, from which we retrieved results of 20 additional original studies, in Table 3 (43 original studies in total). Of the 43 studies, 29 studied mobile apps for reminding, nine SMS reminders and five other types of device alerts. There were 38 RCTs, two non-randomised and three studies using a pre-post design (reported also as case-series and interrupted time series). Twenty-two studies were conducted in the USA and Canada, eleven in Asia, five in Europe, three in Africa and two in Australia. Twelve studies reported participants’ mean age above 60 years, whereas six reported the mean age below 50, including only a small share of participants aged 65 and older. Four original studies did not allow for the comparison of mean ages. Study and intervention characteristics together with all outcome results are described in more detail in Appendix Tables A3–A4.

**Table 2:** Results of methodological quality assessment: Medication reminder original studies.

Study	Randomisation/Representativeness	Similar at baseline/Appropriate measurements	Complete outcome data/Low non-response bias risk	Blinding of assessors/Confounding accounted for	Adherence to intervention/Exposure as intended/Strategy	Total
Bediang [20]	1	1	0	1	0	3/5
Buis [21]	1	0	1	0	1	3/5
Criner [37]	0	0	1	0	0	1/5
Dıgın [36]	1	0	1	0	1	3/5
Farmer [19]	1	1	1	1	1	5/5
Graetz [38]	1	1	0	0	1	3/5
Greer [18]	1	1	0	0	1	3/5
Habib [61]	1	0	1	1	0	3/5
Huang [28]	1	1	0	1	0	3/5
Li [30]	1	1	0	1	0	3/5
Marvel [62]	0	1	1	1	1	4/5
Mauro [63]	0	1	1	0	0	2/5
McGilliguddy [31]	0	1	1	1	0	3/5
Morawski [23]	1	1	1	0	1	4/5
Movva [33]	0	0	1	0	1	2/5
Ni/2018 [26]	1	0	1	0	0	2/5
Ni/2022 [27]	1	0	1	0	0	2/5
Ong [32]	1	1	0	0	0	2/5
Ravari [22]	0	1	1	0	1	3/5
Santo [25]	1	0	1	1	1	4/5
Sikorskii [35]	1	1	1	1	0	4/5
Solmaz [24]	0	1	1	0	1	3/5
van de Hei [34]	1	1	0	0	0	2/5

MMAT questions 1–5 by study type: all are RCTs except Marvel [62], which is a quantitative non-randomised study.

**Table 3:** Results of quality assessment: Medication reminder systematic reviews.

	Al-Arkee [39]	Aung [59]	Choi [45]	Cross [64]	Ng [49]	Redfern [50]/Adler [51]	Stevenson [56]	Yap [65]
PICO components	1	1	1	1	1	1	1	1
Protocol or prior methods	½	1	½	1	0	1	1	1
RCT/NRSI selection explained	0	1	0	0	0	1	0	0
Comprehensive search strategy	1	½	½	1	½	1	1	½
Duplicate study selection	0	1	1	1	0	1	1	1
Duplicate data extraction	0	1	0	1	0	1	1	1
List of excluded studies	0	0	0	1	0	1	1	0
Studies described in detail	½	1	1	1	0	1	1	½
Satisfactory RoB technique	1	1	1	1	1	1	1	1
Reported on funding of studies	0	0	0	1	1	1	1	0
MA: appropr. Stat. methods	0	N	N	1	N	N	N	N
MA: RoB impact of studies	0	N	N	1	N	N	N	N
RoB of studies in result interpret.	0	0	0	1	1	1	1	1
Result heterogeneity discussed	0	0	0	1	1	1	1	1
MA: publication bias analysis	0	N	N	0	N	N	N	N
Reported on conflict of interest	1	1	1	1	1	1	1	1
Total:	5/16	8½/16	6/16	14/16	6½/16	13/16	12/16	9/16

16 AMSTAR2 questions (answered through our extracted results). Yes = 1, Partial Yes = ½, No = 0, MA = Meta-analysis, N = No meta-analysis conducted. Evaluations for Redfern *et al*. (2024), an update of Adler *et al*. (2017).

### Study quality and risk of bias

Quality ratings of the original studies and systematic reviews are displayed in Tables 2 and 3. As the systematic reviews reported clinical health effects in the original studies, we applied the evaluation criteria to these outcomes, omitting adherence and other intermediate outcomes.

### Outcomes of medication reminder technologies

#### Care experiences of older adults and carers

One original study [18] examined the effects of mobile apps for reminding on patients’ satisfaction with clinician explanations, interpersonal treatment, care comprehensiveness, nursing communication and trust and confidence in clinicians. The effects obtained from the pre-post study design were not statistically significant for any of the satisfaction measures. Also two other studies, reporting on satisfaction with health care [19] and general management of patients [20], found no significant differences between the intervention and control groups although the results slightly favoured the SMS intervention. Studies that were included in the systematic reviews did not report results regarding patient satisfaction. Considering the lack of significance and consistency of the results, we conclude that there is no evidence of beneficial effects of reminders on patient satisfaction with care. Furthermore, we found no studies evaluating the effects of medication reminders on carer’s experiences.

#### Wellbeing and experiences of care professionals

We found no studies examining the effects of medication reminders on care professionals’ experiences or wellbeing.

#### Health and/or wellbeing outcomes

Most studies reported on health outcomes, either self-reported or clinical outcomes. To ease their interpretation, we describe the results separately for original studies and systematic reviews, and by subcategories of health outcomes, which are listed in Table 4.

**Table 4:** Main results of health outcomes from medication reminder interventions

Outcome	Number of studies with significant findings out of total studies on outcome categories	
BP, cholesterol, HbA1C	12/21	Brath [40], Patel [48], Mira [53], Davidson [46], Albini [47], Frias [42], Liu [43], Chandler [41], Ravari [22], Farmer [19], Li [30], Ni/2022 [27]
Other clinical outcomes	4/16	Feng [29], Ni/2018 [26], McGillicuddy [31], Movva [33]
Symptoms	3/5	Khonsari [57], Sikorskii [35], Graetz [38]
Quality of life	2/10	Graetz [38], van de Hei [34]

*Notes:* Ni/2018 [26] heart rate result has non-beneficial direction. QoL category also includes SAI, EQ-5D, CCQ, ACQ-5, anxiety, cognitive and self-perceived health.

### Original studies

#### Blood pressure, cholesterol and HbA1c

Four original studies focused on hypertension patients [21–24]. The improvement in systolic blood pressure (SBP) and diastolic blood pressure (DBP) caused by SMS reminders in the 1-month follow-up was non-significant in one study [21], whereas another study found significant effects of an SMS intervention on DBP in the 1-month follow-up and on SBP in the 2- and 3-month follow-ups [22]. No significant differences in the mean SBP and controlled (<140/90 mmHg) blood pressure (BP) were found between participants using a mobile reminder app and controls [23]. The fourth study found medication reminder wristwatches together with training to improve SBP and DBP compared to two controls (training without wristwatch and no intervention) but pre-post findings show significant improvements in BP outcomes also for the two control groups [24]. We retrieved three studies on mobile reminder app studies used by coronary heart disease patients [25–27]. One study found no significant differences in SBP, DBP and cholesterol levels between mobile reminder app users and the receivers of usual care [25]. Two studies examined the effectiveness of reminder apps with shorter (30 days) and longer (90 days) follow-ups. For SBP and DBP outcomes, findings for the longer follow-up indicated significant differences in the rates of change between the intervention and control groups favouring the intervention [27], whereas for the shorter follow-up, these between-group differences were not statistically significant [26]. Two original studies focused on type 2 diabetes patients [28, 29]. One [28] studied the effectiveness of mobile reminder apps and found no significant differences between treatment and control groups in HbA1c and cholesterol outcomes. The other study [29] found that reminder apps had beneficial effects on SBP, 10-year risk of coronary heart disease, the likelihood of participants meeting treatment goals, HbA1c, cholesterol and health status; only the effects on SBP and the likelihood of meeting treatment goals were significant. A study focusing on polypharmacy patients [30] found statistically significant and beneficial effects for reminder apps, on the likelihood of HbA1c being below 6.5%, total cholesterol (TC) being less than 5.2 mmol/L, and low density lipoprotein cholesterol (LDL-C) being lower in the treatment group than in the control group, whereas their effects on blood glucose (fasting and non-fasting), creatinine, thyroid function test, BP and weight change were all non-significant.

#### Other clinical outcomes

A study focusing on kidney transplant recipients [31] found mobile reminders together with medication tray alerts to significantly improve tacrolimus control. Another study [20] found no significant change in treatment success in curing tuberculosis patients using SMS medication reminders. An original study focusing on the use of mobile reminder apps among coronary heart disease patients [26] reported a significant difference in the pre-post changes of the heart rate between the intervention and control groups. The result favoured the control group, but the intervention group started from a significantly lower baseline level. One original study [32] found no significant differences in several clinical outcomes between users of two apps for organising chronic kidney disease patient care in connection with manual dispensers, lists of medications or nothing. One original study [33] found a significant effect on 1-week visual acuity of cataract surgery patients due to a mobile app but the effect did not remain beyond the second week of the 6-week follow-up, and effects on complication rates (dry eye, cystoid macular edema, posterior capsule opacification) were not significant. One original study found no significant effect on asthma exacerbations of using mobile apps [34].

#### Symptoms

Two studies focusing on oral therapy cancer patients examined the effects of medication reminders on symptom severity. Automated reminder calls had significant beneficial effects after 8-week follow-up, but they did not remain until the 12-week follow-up [35]. Contrarily, a mobile app intervention had no significant beneficial effect, but it was noted that it may help symptoms of those with certain risk factors, such as difficulties with adherence or elevated anxiety [18].

#### Quality of life

The same mobile app intervention [18] was also examined for its impact on quality of life, but no significant effect was found. Another mobile app study targeting type 2 diabetes patients [19] found no significant differences in health status between the intervention and control groups. A study [36] on the effectiveness of SMS reminders in cataract surgery patients found that the change in the State Anxiety Inventory score in the intervention group did not differ significantly from that of the control group. One study [37] found no beneficial effects on the Clinical COPD Questionnaire (CCQ) score (symptoms, functional and mental state) due to medication reminders through an inhaler monitoring device and smartphone app in patients with chronic obstructive pulmonary disease (COPD). One study [38] found significant beneficial effects on mental health and symptoms of SMS reminders together with smart pill bottles in women with breast cancer prescribed capecitabine. Another study [34] found that mobile app reminders significantly improved asthma control (ACQ-5) but found no significant differences in work productivity indicators (absenteeism, presenteeism, and overall activity impairment).

### Systematic reviews

#### Blood pressure, cholesterol and HbA1c

In one review focusing on cardiovascular disease patients [39], three studies (of the seven that met our criteria) found significant effects of mobile app reminders on SBP and DBP, in favour of the app intervention [40–42]. In the same review, cholesterol was assessed as a health outcome in four studies [25, 40, 42, 43], of which three [40, 42, 43] found significant effects of reminder apps on TC and one on LDL-C [42], all favouring the intervention. No significant effects of reminders on HbA1c were found in the two studies that assessed it [40, 42]. Evidence from a meta-analysis in the same review [39] favoured the intervention for the TC and LDL-C [25, 43] and SBP [23, 25, 44] outcomes, but not for the DBP outcome [25, 44]; however, none of the overall effects in this meta-analysis provided significant results. For the same meta-analysis, using 3-month follow-up data only, the review [39] also reported individual results, which were significant only for TC [43] favouring the intervention.

In one review with hypertension and ischemic heart disease patients as target groups [45], two studies [46, 47] found significant differences between the mobile app and control groups in maintaining BP below target levels. One original study [48], included in two reviews [45, 49], found mobile reminder apps to decrease SBP significantly in a pre-post-test design but did not find similar differences for DBP. Two reviews focusing on heart disease patients [50, 51] reported non-significant effects of one study on DBP [52], favouring the SMS intervention group. Based on the findings of an original study with a pre-post-test design [53], one review [49] reported a significant beneficial effect of the mobile reminder app on cholesterol level of patients (no specific medical condition). Two reviews [45, 49] reported findings of two original studies that found no significant effects of mobile reminder apps on levels of SBP, DBP [53, 54], glycated haemoglobin [53], cholesterol and triglyceride among hypertension and/or dyslipidemia patients [54]. A significant reduction in SBP and DBP from the use of a BP self-monitoring app providing active feedback was found in a study focusing on chronic kidney disease patients [55], reported in one systematic review [56]. Applying our evidence levels, we found moderate evidence of beneficial effects of mobile reminder apps on SBP. Regarding DBP, HbA1c and cholesterol levels, we found only limited evidence.

#### Other clinical outcomes

One RCT targeting cardiovascular disease patients [43], reported in one systematic review [39], and another RCT targeting chronically ill (a great majority with hypertension and/or dyslipidemia) patients [54], reported in two systematic reviews [45, 49], found no significant changes in the mean triglyceride levels of using mobile reminder apps. One of these reviews [49] reported results of an endoscopic sinus surgery study [29], finding that the granulation score increased significantly more in the control group than in the mobile app intervention group. Based on an original study [57], two reviews [50, 51] reported zero deaths in the SMS reminder intervention group compared to two deaths in the control group due to acute coronary syndrome complications, but this result lacked statistical significance.

#### Symptoms

The same reviews [50, 51] mentioned also related results [57] showing significant beneficial effects of SMS reminders on heart function status. One review [56] reported reductions in the number of rejection episodes after exposure to electronic monitoring dispenser trays and a SIM-pill system with reminders in comparison to the control condition without reminders [58]. Using our evidence levels, we found moderate evidence of beneficial effects of reminder technologies on physical symptoms.

#### Quality of life

Three reviews [45, 49, 59] reported on quality of life measures, finding no significant effects of medication reminders on the COPD Assessment Test (CAT) score [60], quality of life or EQ-5D [54] or self-perceived health status [53]. Thus, we found no evidence of beneficial effects of medication reminders on quality of life.

### Service utilisation, costs and cost-effectiveness

#### Service use outcomes

Three original studies [18, 61, 62] examined the effects of mobile reminder apps on service use. In general, the direction of the intervention effect was as expected, i.e. pointing to a lower use of services. The first study [18] found no significant effect of a mobile reminder app on emergency department (ED) visits or hospitalisations, though both favoured the intervention. With no statistical testing, the second study [61] reported lower ED visits in the mobile reminder app group compared to the control group, but for re-hospitalisations the findings were reversed favouring the control group. The third study [62] found the risk of re-hospitalisations 30-days post-discharge to be lower (−52%) in the intervention group (patients with a mobile app, smartwatch and BP-monitor) than in the control group. This study also tested the effects of the intervention on ED visits (excluding patients that were hospitalised afterwards) and found that controls had less likely ED visits, but the difference with the intervention group was not significant. One original study [57], reported in two systematic reviews [50, 51] found SMS reminders to reduce hospital readmissions but the effect was non-significant. Based on these results, there is limited evidence of beneficial effects of reminder technologies on health service use.

#### Costs and cost-effectiveness

One original study [63] reported that a smart pill bottle alert intervention resulted in a 12.6 percentage point increase in median adherence compared to a control group in which all SMS and pill bottle alerts were deactivated, with an incremental cost-effectiveness ratio of $96 (annualised) per one percentage point increase in adherence. One original study [34] compared inhalator use together with mobile app reminders to passively monitored inhaler use and reported that the cost per 0.5 point (the minimal clinically important difference) decrease in ACQ-5 due to reminders was €278 for one year.

### Equity

None of the included studies evaluated the effects of medication reminder technologies on equity.

## Discussion

### Summary of the evidence

Of the 43 original studies, 40 assessed health outcomes, with 20 showing significant beneficial effects. There is moderate evidence that medication reminder technologies improve SBP and reduce physical symptoms. For other outcomes there is limited or no evidence. Improvements in SBP were more consistent in app-based studies compared to SMS-based studies, although both varied depending on the specific population and setting. For physical symptoms, the other outcome for which moderate evidence was found, app-based technologies did not seem superior to SMS reminders in improving physical symptoms, but effects depend on condition, co-components and follow-up.

We found no indication that beneficial effects of medication reminders were more common in studies using a particular study design, conducted in a specific geographical area, or within a certain patient age group. Age and age-varying effects were hardly reported; only two studies reported age interactions with treatment results of mobile reminder apps for clinical health outcomes [19, 23].

### Strengths and limitations of the review

We studied the effects of medication reminder technologies, excluding studies in which the technology was part of a broader, multi-component intervention, which enabled us to get more precise results regarding the effects of reminders. Furthermore, we excluded studies that exclusively reported medication adherence outcomes, considering that for informed decision-making on the usefulness of implementing these technologies to support independent living and reducing the burden on health services the evidence should go beyond the effects on medication adherence and address broader Quintuple Aim outcomes. We also used a more comprehensive systematic review approach by screening the literature with broader search terms to avoid false exclusions of studies. Our comprehensive approach also enabled us to synthesise the results of various study types, including original studies and quality assessments from previous systematic reviews, scaled to common evidence levels.

### Recommendations for practice and research

Medication reminder technologies, which with the spread of mobile phones are nowadays usually reminder apps, present an enticing low-entry solution to support medication management among the home-dwelling ageing population. Policymakers should consider supporting the integration of low-cost reminder technologies into national ageing strategies, particularly in home-based care and community support services. Given their scalability and low entry cost, such technologies may be particularly valuable in low- and middle-income countries, where access to formal care is limited. However, the current evidence of their effectiveness is still limited, showing improvement in selected clinical outcomes only. Nevertheless, improvement of clinical outcomes among ageing population is highly important, as better health may contribute substantially to wellbeing and independent living, which at their turn may reduce the pressure on countries’ health and care services. Our review highlights the need for more high-quality studies that assess the effects of using medication reminders in daily life on the use of health and care services, cost-effectiveness, care professionals’ workload and wellbeing, and equity indicators as well as for subgroup analysis for various ages and population groups living in remote areas or with limited digital skills and literacy.

## Conclusion

There is moderate evidence that the use of medication reminders improves certain clinical health outcomes among community-dwelling older citizens, but no or only limited evidence that it could also improve outcomes within other Quintuple Aim domains. More high-quality studies are needed to build the evidence; only then the potential of medication technologies to contribute to solving some of the challenges related to population ageing could be evaluated on its true merits.

## Supplementary Material

afag007_Supplemental_File

## Data Availability

Supporting online supplement is available.
