# Supplementary material for: Effects of using medication reminder technologies by home-dwelling older citizens: a systematic review
Source: Age Ageing. 2026 Feb 5;55(2):afag007. doi: 10.1093/ageing/afag007 (PMC13008587; doi:10.1093/ageing/afag007)
Supplement: afag007_Supplemental_File [file afag007_supplemental_file.docx]

**Appendix Table A1. PRISMA 2020 Checklist^†^**

| **Section and Topic** | **Item #** | **Checklist item** | **Location/section of the article where item is reported** |
| --- | --- | --- | --- |
| **TITLE**  **Effects of using medication reminder technologies by home-dwelling older citizens: A systematic review** | | |  |
| Title | 1 | Identify the report as a systematic review. | Title |
| **ABSTRACT** | | |  |
| Abstract | 2 | See the PRISMA 2020 for Abstracts checklist. | Abstract |
| **INTRODUCTION** | | |  |
| Rationale | 3 | Describe the rationale for the review in the context of existing knowledge. | Background and rationale for the review |
| Objectives | 4 | Provide an explicit statement of the objective(s) or question(s) the review addresses. | Objective of the review |
| **METHODS** | | |  |
| Eligibility criteria | 5 | Specify the inclusion and exclusion criteria for the review and how studies were grouped for the syntheses. | Study selection |
| Information sources | 6 | Specify all databases, registers, websites, organisations, reference lists and other sources searched or consulted to identify studies. Specify the date when each source was last searched or consulted. | Search strategy and data sources |
| Search strategy | 7 | Present the full search strategies for all databases, registers and websites, including any filters and limits used. | Search strategy and data sources |
| Selection process | 8 | Specify the methods used to decide whether a study met the inclusion criteria of the review, including how many reviewers screened each record and each report retrieved, whether they worked independently, and if applicable, details of automation tools used in the process. | Data extraction and quality assessment |
| Data collection process | 9 | Specify the methods used to collect data from reports, including how many reviewers collected data from each report, whether they worked independently, any processes for obtaining or confirming data from study investigators, and if applicable, details of automation tools used in the process. | Data extraction and quality assessment |
| Data items | 10a | List and define all outcomes for which data were sought. Specify whether all results that were compatible with each outcome domain in each study were sought (e.g. for all measures, time points, analyses), and if not, the methods used to decide which results to collect. | Study selection; Appendix tables A3-A4 |
|  | 10b | List and define all other variables for which data were sought (e.g. participant and intervention characteristics, funding sources). Describe any assumptions made about any missing or unclear information. | Study and intervention characteristics; Appendix tables A3-A4 |
| Study risk of bias assessment | 11 | Specify the methods used to assess risk of bias in the included studies, including details of the tool(s) used, how many reviewers assessed each study and whether they worked independently, and if applicable, details of automation tools used in the process. | Data extraction and quality assessment |
| Effect measures | 12 | Specify for each outcome the effect measure(s) (e.g. risk ratio, mean difference) used in the synthesis or presentation of results. | Appendix tables A3-A4 |
| Synthesis methods | 13a | Describe the processes used to decide which studies were eligible for each synthesis (e.g. tabulating the study intervention characteristics and comparing against the planned groups for each synthesis (item #5)). | NA |
|  | 13b | Describe any methods required to prepare the data for presentation or synthesis, such as handling of missing summary statistics, or data conversions. | NA |
|  | 13c | Describe any methods used to tabulate or visually display results of individual studies and syntheses. | NA |
|  | 13d | Describe any methods used to synthesize results and provide a rationale for the choice(s). If meta-analysis was performed, describe the model(s), method(s) to identify the presence and extent of statistical heterogeneity, and software package(s) used. | Data extraction and quality assessment, Synthesis of results and evidence levels |
|  | 13e | Describe any methods used to explore possible causes of heterogeneity among study results (e.g. subgroup analysis, meta-regression). | Discussion |
|  | 13f | Describe any sensitivity analyses conducted to assess robustness of the synthesized results. | Discussion |
| Reporting bias assessment | 14 | Describe any methods used to assess risk of bias due to missing results in a synthesis (arising from reporting biases). | Data extraction and quality assessment |
| Certainty assessment | 15 | Describe any methods used to assess certainty (or confidence) in the body of evidence for an outcome. | Data extraction and quality assessment, Synthesis of results and evidence levels |
| **RESULTS** | | |  |
| Study selection | 16a | Describe the results of the search and selection process, from the number of records identified in the search to the number of studies included in the review, ideally using a flow diagram. | Search strategy and data sources, Figure 1 |
|  | 16b | Cite studies that might appear to meet the inclusion criteria, but which were excluded, and explain why they were excluded. | Appendix table A2 |
| Study characteristics | 17 | Cite each included study and present its characteristics. | Appendix tables A3-A4 |
| Risk of bias in studies | 18 | Present assessments of risk of bias for each included study. | Study and intervention characteristics, Study quality and risk of bias, Tables 1-3 |
| Results of individual studies | 19 | For all outcomes, present, for each study: (a) summary statistics for each group (where appropriate) and (b) an effect estimate and its precision (e.g. confidence/credible interval), ideally using structured tables or plots. | Appendix tables A3-A4 |
| Results of syntheses | 20a | For each synthesis, briefly summarise the characteristics and risk of bias among contributing studies. | Study and intervention characteristics, Study quality and risk of bias |
|  | 20b | Present results of all statistical syntheses conducted. If meta-analysis was done, present for each the summary estimate and its precision (e.g. confidence/credible interval) and measures of statistical heterogeneity. If comparing groups, describe the direction of the effect. | Summary of the evidence, Appendix tables A3-A4 |
|  | 20c | Present results of all investigations of possible causes of heterogeneity among study results. | Appendix tables A3-A4 |
|  | 20d | Present results of all sensitivity analyses conducted to assess the robustness of the synthesized results. | Appendix tables A3-A4 |
| Reporting biases | 21 | Present assessments of risk of bias due to missing results (arising from reporting biases) for each synthesis assessed. | Study and intervention characteristics, Study quality and risk of bias, Tables 1-3 |
| Certainty of evidence | 22 | Present assessments of certainty (or confidence) in the body of evidence for each outcome assessed. | Summary of the evidence |
| **DISCUSSION** | | |  |
| Discussion | 23a | Provide a general interpretation of the results in the context of other evidence. | Summary of the evidence |
|  | 23b | Discuss any limitations of the evidence included in the review. | Strengths and limitations of the review |
|  | 23c | Discuss any limitations of the review processes used. | Strengths and limitations of the review |
|  | 23d | Discuss implications of the results for practice, policy, and future research. | Recommendations for practice and research |
| **OTHER INFORMATION** | | |  |
| Registration and protocol | 24a | Provide registration information for the review, including register name and registration number, or state that the review was not registered. | NA |
|  | 24b | Indicate where the review protocol can be accessed, or state that a protocol was not prepared. | NA |
|  | 24c | Describe and explain any amendments to information provided at registration or in the protocol. | NA |
| Support | 25 | Describe sources of financial or non-financial support for the review, and the role of the funders or sponsors in the review. | Funding |
| Competing interests | 26 | Declare any competing interests of review authors. | Acknowledgements |
| Availability of data, code and other materials | 27 | Report which of the following are publicly available and where they can be found: template data collection forms; data extracted from included studies; data used for all analyses; analytic code; any other materials used in the review. | Appendix tables A3-A4 |

^†^Page MJ, McKenzie JE, Bossuyt PM, Boutron I, Hoffmann TC, Mulrow CD, et al. The PRISMA 2020 statement: An updated guideline for reporting systematic reviews. *BMJ (Online)*, *372*, n71–n71. <https://doi.org/10.1136/bmj.n71>

**Appendix Table A2. Excluded studies with reasons.**

1. Abaza H; Marschollek M. SMS education for the promotion of diabetes self-management in low & middle income countries: a pilot randomized controlled trial in Egypt. BMC Public Health Dec 2017;17(1):962. DOI: 10.1186/s12889-017-4973-5. **Wrong intervention**
2. Ahmad A; Chiu V; Arain MA. Users' Perceptions of an in-Home Electronic Medication Dispensing System: A Qualitative Study. Med Devices (Auckl) 2020;13():31-39. DOI: 10.2147/MDER.S241062. **Wrong comparator**
3. Akhu-Zaheya, L.M.; Shiyab, W.Y. The effect of short message system (SMS) reminder on adherence to a healthy diet, medication, and cessation of smoking among adult patients with cardiovascular diseases. International Journal of Medical Informatics 2017;98():65-75. DOI: 10.1016/j.ijmedinf.2016.12.003. **Wrong outcomes**
4. Anghel, I.; Cioara, T.; Moldovan, D.; Antal, M.; Pop, C.D.; Salomie, I.; Pop, C.B.; Chifu, V.R. Smart environments and social robots for age-friendly integrated care services. International Journal of Environmental Research and Public Health 2020;17(11). DOI: 10.3390/ijerph17113801. **Wrong study design**
5. Anstey Watkins JOT; Goudge J; Gómez-Olivé FX; Griffiths F. Mobile phone use among patients and health workers to enhance primary healthcare: A qualitative study in rural South Africa. Soc Sci Med Feb 2018;198():139-147. DOI: 10.1016/j.socscimed.2018.01.011. **Wrong intervention**
6. Armitage, L.C.; Kassavou, A.; Sutton, S. Do mobile device apps designed to support medication adherence demonstrate efficacy? A systematic review of randomised controlled trials, with meta-analysis. BMJ Open 2020;10(1). DOI: 10.1136/bmjopen-2019-032045. **Wrong outcomes**
7. Asghar, I; Cang, S; Yu, HN. The impact of assistive software application to facilitate people with dementia through participatory research. International Journal of Human-Computer Studies 2020;143. DOI: 10.1016/j.ijhcs.2020.102471. **Wrong intervention**
8. Bailey, SC; Wismer, GA; Parker, RM et al. Development and rationale for a multifactorial, randomized controlled trial to test strategies to promote adherence to complex drug regimens among older adults. Contemporary Clinical Trials 2017; 62; 21‐26. DOI: 10.1016/j.cct.2017.08.013. **Wrong study design**
9. Balasubramanian GV; Beaney P; Chambers R. Digital personal assistants are smart ways for assistive technology to aid the health and wellbeing of patients and carers. BMC Geriatr Nov 2021;21(1):643. DOI: 10.1186/s12877-021-02436-y. **Wrong comparator**
10. Bauer KL et al. Improving follow-up attendance for discharged emergency care patients. Academic Emergency Medicine 2021, 28(2). DOI: 10.1111/acem.14080. **Wrong intervention**
11. Barrué, C.; Vila, R.; Martinez, A.B.; Martín, M.; Vázquez-Salceda, J.; Simó, R.; Barroso, M.; Cortés, U. DIALCAT: Diabetes as an accelerator of cognitive impairment and Alzheimer's disease, comprehensive approach and adherence to treatment. Computacion y Sistemas 2019;23(2):351-363. DOI: 10.13053/CyS-23-2-3198. **Wrong outcomes**
12. Baxter, M.S.; Tibble, H.; Bush, A.; Sheikh, A.; Schwarze, J. Effectiveness of mobile health interventions to improve nasal corticosteroid adherence in allergic rhinitis: A systematic review. Clinical and Translational Allergy 2021;11(9). DOI: 10.1002/clt2.12075. **Wrong patient population**
13. Behera, C.K.; Condell, J.; Dora, S.; Gibson, D.S.; Leavey, G. State-of-the-Art Sensors for Remote Care of People with Dementia during a Pandemic: A Systematic Review. Sensors 2021, 21(14):4688. https://doi.org/10.3390/s21144688. **Wrong comparator**
14. Berry DL; Blonquist TM; Nayak MM; Grenon N; Momani TG; McCleary NJ. Self-Care Support for Patients with Gastrointestinal Cancer: iCancerHealth. Appl Clin Inform Oct 2018;9(4):833-840. DOI: 10.1055/s-0038-1675810. **Wrong comparator**
15. Bolaños, M.; Collazos, C.; Gutiérrez, F. Adapting a virtual assistant device to support the interaction with elderly people. TEG 2020 - Special Session on Technology, Elderly Games; 285-292. **Wrong comparator**
16. Boonnuddar, N.; Wuttidittachotti, P. Mobile application: Patients' adherence to medicine in-take schedules. BDIOT 2017; 237-241. DOI: 10.1145/3175684.3175714. **Wrong study design**
17. Buis LR; Roberson DN; Kadri R; Rockey NG; Plegue MA; Danak SU; Guetterman TC; Johnson MG; Choe HM; Richardson CR. Understanding the Feasibility, Acceptability, and Efficacy of a Clinical Pharmacist-led Mobile Approach (BPTrack) to Hypertension Management: Mixed Methods Pilot Study. J Med Internet Res Aug 2020;22(8):e19882. DOI: 10.2196/19882. **Wrong patient population**
18. Casciaro, S.; Massa, L.; Sergi, I.; Patrono, L. A Smart Pill Dispenser to support Elderly People in Medication Adherence. 2020, 5th International Conference on Smart and Sustainable Technologies (SpliTech). DOI: 10.23919/SpliTech49282.2020.9243773. **Wrong outcomes**
19. Chan A, De Simoni A, Wileman V, Holliday L, Newby CJ, Chisari C, Ali S, Zhu N, Padakanti P, Pinprachanan V, Ting V, Griffiths CJ. Digital interventions to improve adherence to maintenance medication in asthma. Cochrane Database of Systematic Reviews 2022, Issue 6. Art. No.: CD013030. DOI: 10.1002/14651858.CD013030.pub2. **Wrong intervention**
20. Chaturvedi A, Mathur G, Maheshwari S, Samaria A, Rijhwani P, Patidar P, Singh N, Kumar V, Kumar S, Gwalani HD, et al. Impact of mobile health application use among geriatric diabetes mellitus patients: a multicentric study from Rajasthan, India. Diabetes Technology & Therapeutics, 2025; 27(S2): e315‐e316. https://doi.org/10.1089/dia.2024.78502.abstracts. **Wrong study design**
21. Chavez, E.; Sifuentes, B.; Vidal, R.; Grados, J.; Rubinõs, S.; Cuzcano, A. Remote Monitoring Applying IoT to Improve Control of Medication Adherence in Geriatric Patients with a complex Treatment Regimen, Lima-Peru. EEET '20: Proceedings of the 2020 3rd International Conference on Electronics and Electrical Engineering Technology. September 2020, 49-54. DOI: 10.1145/3429536.3429543. **Wrong outcomes**
22. Cho, M.-K.; Kim, M.Y. Self-management nursing intervention for controlling glucose among diabetes: A systematic review and meta-analysis. International Journal of Environmental Research and Public Health 2021;18(23). DOI: 10.3390/ijerph182312750. **Wrong intervention**
23. Choi, W.S.; Choi, J.H.; Oh, J.; Shin, I.-S.; Yang, J.-S. Effects of Remote Monitoring of Blood Pressure in Management of Urban Hypertensive Patients: A Systematic Review and Meta-Analysis. Telemedicine and e-Health 2020;26(6):744-759. DOI: 10.1089/tmj.2019.0028. **Wrong intervention**
24. Christiansen, L.; Lindberg, C.; Sanmartin Berglund, J.; Anderberg, P.; Skär, L. Using mobile health and the impact on health-related quality of life: Perceptions of older adults with cognitive impairment. International Journal of Environmental Research and Public Health 2020;17(8). DOI: 10.3390/ijerph17082650. **Wrong intervention**
25. Christiansen, L.; Sanmartin Berglund, J.; Anderberg, P.; Cellek, S.; Zhang, J.; Lemmens, E.; Garolera, M.; Mayoral-Cleries, F.; Skär, L. Associations Between Mobile Health Technology use and Self-rated Quality of Life: A Cross-sectional Study on Older Adults with Cognitive Impairment. Gerontology and Geriatric Medicine 2021;7. DOI: 10.1177/23337214211018924. **Wrong intervention**
26. Ciampi, M.; Coronato, A.; Naeem, M.; Silvestri, S. An intelligent environment for preventing medication errors in home treatment. Expert Systems with Applications 2022;193. DOI: 10.1016/j.eswa.2021.116434. **Wrong setting**
27. Czaja, SJ. The role of technology in supporting healthcare for older adults. International Society for Gerontechnology's (ISG) 12th World Conference of Gerontechnology, October 6-9, 2020 (Virtual), 19; 80. DOI: 10.4017/gt.2020.19.s.69681.3. **Wrong intervention**
28. Dang TH, Wickramasinghe N, Jayaraman PP, Burbury K, Alexander M, Whitechurch A, Dyer M, Quinn S, Forkan ARM, Schofield P. Digital Solution to Support Medication Adherence and Self-Management in Patients with Cancer (SAMSON): Pilot Randomized Controlled Trial. JMIR Form Res 2025;9:e65302. doi: 10.2196/65302. **Wrong intervention**
29. de Barra, M; Scott, CL; Scott, NW; Johnston, M; de Bruin, M; Nkansah, N; Bond, CM; Matheson, CI; Rackow, P; Williams, AJ; et al. Pharmacist services for non‐hospitalised patients. Cochrane Database of Systematic Reviews 2018;(9); John Wiley & Sons, Ltd 2018. DOI: 10.1002/14651858.CD013102. **Wrong intervention**
30. Debon R; Coleone JD; Bellei EA; De Marchi ACB. Mobile health applications for chronic diseases: A systematic review of features for lifestyle improvement. Diabetes Metab Syndr Jul-Aug 2019;13(4):2507-2512. DOI: 10.1016/j.dsx.2019.07.016. **Wrong intervention**
31. Dewani, A.; Lakho, M.R.; Haider Buledi, G.; Samoo, U.; Anwer, N.; Latif, M.; Shaikh, A. DemCure: A Technology-oriented solution for Assisting Caretakers and Dementia Patients with Mild Cognitive Impairment. 2020 3rd International Conference on Computing, Mathematics and Engineering Technologies – iCoMET 2020. DOI: 10.1109/iCoMET48670.2020.9074142. **Wrong study design**
32. Dobson R; Whittaker R; Jiang Y; Maddison R; Shepherd M; McNamara C; Cutfield R; Khanolkar M; Murphy R. Effectiveness of text message based, diabetes self management support programme (SMS4BG): two arm, parallel randomised controlled trial. BMJ May 2018;361. DOI: 10.1136/bmj.k1959. **Wrong intervention**
33. Donevant, S.B.; Estrada, R.D.; Culley, J.M.; Habing, B.; Adams, S.A. Exploring app features with outcomes in mHealth studies involving chronic respiratory diseases, diabetes, and hypertension: A targeted exploration of the literature. Journal of the American Medical Informatics Association 2018;25(10):1407-1418. DOI: 10.1093/jamia/ocy104. **Wrong study design**
34. Drabarek D; Anh NT; Nhung NV; Hoa NB; Fox GJ; Bernays S. Implementation of Medication Event Reminder Monitors among patients diagnosed with drug susceptible tuberculosis in rural Viet Nam: A qualitative study. PLoS One 2019;14(7):e0219891. DOI: 10.1371/journal.pone.0219891. **Wrong patient population**
35. Eggerth, A.; Hayn, D.; Schreier, G. Medication management needs information and communications technology-based approaches, including telehealth and artificial intelligence. British Journal of Clinical Pharmacology 2020;86(10):2000-2007. DOI: 10.1111/bcp.14045. **Wrong study design**
36. El-Saifi, N.; Moyle, W.; Jones, C.; Tuffaha, H. Medication Adherence in Older Patients With Dementia: A Systematic Literature Review. Journal of Pharmacy Practice 2018;31(3):322-334. DOI: 10.1177/0897190017710524. **Wrong intervention**
37. Ernsting C; Dombrowski SU; Oedekoven M; O Sullivan JL; Kanzler M; Kuhlmey A; Gellert P. Using Smartphones and Health Apps to Change and Manage Health Behaviors: A Population-Based Survey. J Med Internet Res Apr 2017;19(4):e101. DOI: 10.2196/jmir.6838. **Wrong intervention**
38. EUCTR2016-003986-25-ES. Impact of self-measurement of blood pressure and self-adjustment of antihypertensive medication in the control of hypertension and adherence to treatment. A pragmatic, randomized, controlled clinical trial (ADAMPA Study), 2017. **Wrong study design**
39. Faisal, S.; Ivo, J.; McDougall, A.; Patel, T. Stakeholder feedback of electronic medication adherence products: Qualitative analysis. Journal of Medical Internet Research 2020;22(12). DOI: 10.2196/18074. **Wrong comparator**
40. Fallah M; Yasini M. A Medication Reminder Mobile App: Does It Work for Different Age Ranges. Stud Health Technol Inform 2017;235():68-72. **Wrong outcomes**
41. Foley, L.; Larkin, J.; Lombard-Vance, R.; Murphy, A.W.; Hynes, L.; Galvin, E.; Molloy, G.J. Prevalence and predictors of medication non-Adherence among people living with multimorbidity: A systematic review and meta-Analysis. BMJ Open 2021;11(9). DOI: 10.1136/bmjopen-2020-044987. **Wrong intervention**
42. Foster JM; Reddel HK; Usherwood T; Sawyer SM; Smith L. Patient-perceived acceptability and behaviour change benefits of inhaler reminders and adherence feedback: A qualitative study. Respir Med Aug 2017;129, Aug, 39-45. DOI: 10.1016/j.rmed.2017.05.013. **Wrong comparator**
43. Garmendia, C.M.; Miranda, R.M.P.; Verello, E.; Goyeneche, A.; Furmento, J.F.; Costabel, J.P. Use of a smartphone application to increase adherence to medical treatment. Medicina (Argentina) 2021;81(1):11-15. **Wrong outcomes**
44. George, J; McNamara, K; Jackson, S; Hughes, J; Peterson, G; Bailey, M; Hsueh, A; Laua, R; Bortoloetto, D; Stewart, K. The HAPPY trial: a randomised controlled trial of a community pharmacy-based intervention for improving patient adherence to antihypertensive medicines. 2010; 18: 22‐23. DOI: 10.1111/j.2042-7174.2010.tb00509.x. **Wrong intervention**
45. Geramita EM; DeVito Dabbs AJ; DiMartini AF; Pilewski JM; Switzer GE; Posluszny DM; Myaskovsky L; Dew MA. Impact of a Mobile Health Intervention on Long-term Nonadherence After Lung Transplantation: Follow-up After a Randomized Controlled Trial. Transplantation Mar 2020;104(3):640-651. DOI: 10.1097/TP.0000000000002872. **Wrong outcomes**
46. Goh CC, Gan XM, Klainin-Yobas P. Effectiveness of Digital-Based Interventions on Physical and Psychological Outcomes Among Cancer Patients: A Systematic Review and Meta-Analysis, Semin Oncol Nurs Feb 2025;41(1):151796, https://doi.org/10.1016/j.soncn.2024.151796. **Wrong intervention**
47. González-Bueno, J.; Calvo-Cidoncha, E.; Nieto-Martín, M.D.; Pérez-Guerrero, C.; Ollero-Baturone, M.; Santos-Ramos, B. Selection of interventions aimed at improving medication adherence in patients with multimorbidity. European Journal of Hospital Pharmacy 2019;26(1):39-45. DOI: 10.1136/ejhpharm-2017-001240. **Wrong outcomes**
48. Gotlieb E, Marzoughi S, Kwon C-S, Harmon M, Kimura M, Truesdale A, Sweetnam C, Soudant C, Downes MH, Busis NA. Clinical effectiveness, feasibility, acceptability, and usability of mobile health applications for epilepsy: A systematic review. Epilepsia 2025;66(5):1349-1373. DOI: 10.1111/epi.18288. **Wrong intervention**
49. Graetz I; Anderson JN; McKillop CN; Stepanski EJ; Paladino AJ; Tillmanns TD. Use of a web-based app to improve postoperative outcomes for patients receiving gynecological oncology care: A randomized controlled feasibility trial. Gynecol Oncol Aug 2018;150(2):311-317. DOI: 10.1016/j.ygyno.2018.06.007. **Wrong intervention**
50. Gray, S.L.; Hart, L.A.; Perera, S.; Semla, T.P.; Schmader, K.E.; Hanlon, J.T. Meta-analysis of Interventions to Reduce Adverse Drug Reactions in Older Adults. Journal of the American Geriatrics Society 2018;66(2):282-288. DOI: 10.1111/jgs.15195. **Wrong intervention**
51. Hackett, K; Lehman, S; Divers, R; Ambrogi, M; Gomes, L; Tan, CC; Giovannetti, T. Remind Me To Remember: a pilot study of a novel smartphone reminder application for older adults with dementia and mild cognitive impairment. Neuropsychological Rehabilitation 2022; 32(1): 22‐50. DOI: 10.1080/09602011.2020.1794909. **Wrong setting**
52. Haslinda N, Juni MH. Effectiveness of health education module delivered through WhatsApp to enhance treatment adherence and successful outcome of tuberculosis in Seremban District, Negeri Sembilan, Malaysia. International Journal of Public Health and Clinical Sciences, Vol. 6, No. 4, 2019. https://doi.org/10.32827/ijphcs.6.4.145. **Wrong patient population**
53. Hallewell Haslwanter, JD. Review of telecare technologies for older people. ICCHP 2018, LNCS 10897: 407–415. DOI: 10.1007/978-3-319-94274-2_58. **Wrong study design**
54. Hernández-Pinzón, C.; Flórez-Flórez, ML. Treatment adherence in heart failure and information and communication technologies. Revista Colombiana de Cardiologia 2017;24(2):96-104. DOI: 10.1016/j.rccar.2016.07.001. **Wrong outcomes**
55. Himes, BE; Leszinsky, L; Walsh, R; Hepner, H; Wu, AC. Mobile Health and Inhaler-Based Monitoring Devices for Asthma Management. Journal of Allergy and Clinical Immunology - In Practice 2019;7(8):2535-2543. DOI: 10.1016/j.jaip.2019.08.034. **Wrong study design**
56. Holmstrand EC, Sato H, Li JM, Mukherjee A, Fitzpatrick NE, Rayl KR, Colangelo, FR. Digital hypertension management: clinical and cost outcomes of a pilot implementation of the OMRON hypertension management platform. Frontiers in digital health 2023;5. DOI: 10.3389/fdgth.2023.1128553. **Wrong intervention**
57. Huang, CY; Nguyen, PA; Clinciu, DL; Hsu, CK; Lu, JR; Yang, HC; Wu, CC; Tsai, WC; Chou, YC; Kuo, TBJ; et al. A personalized medication management platform (PMMP) to improve medication adherence: a randomized control trial. Computer Methods and Programs in Biomedicine 2017; 140: 275‐281. DOI: 10.1016/j.cmpb.2016.12.012. **Wrong outcomes**
58. Huerta-Ramos E; Marcó-García S; Escobar-Villegas MS; Rubio-Abadal E; Ochoa S; Grasa Bello EM; Alonso Solís A; Rabella M; Berdun J; Hospedales M; M-Resist G; Corripio I; Usall J. m-RESIST, a complete m-Health solution for patients with treatmentresistant schizophrenia: a qualitative study of user needs and acceptability in the Barcelona metropolitan area. Actas Esp Psiquiatr 2017;45(6):277-89. **Wrong patient population**
59. Hunegnaw MT, Mesinovic J, Jansons P, George ES, De Ross B, Kiss N, Ebeling PR, Daly RM, Gvozdenko E, Scott D. Feasibility and effectiveness of a digital voice assistant for improving anti-osteoporosis medication adherence, and osteoporosis knowledge and attitudes, in postmenopausal women with osteoporosis: A 12-month randomised controlled trial. Arch Osteoporos Apr 2025;20(1):43. DOI: 10.1007/s11657-025-01529-0. **Wrong intervention**
60. Izeogu, C.; Kalinowski, J.; Schoenthaler, A. Strategies to Improve Adherence to Anti-Hypertensive Medications: a Narrative Review. Current Hypertension Reports 2020; 22(12). DOI: 10.1007/s11906-020-01115-4. **Wrong outcomes**
61. Jafarimanesh, H; Matourypour, P; Sadrnia, S; Ranjbaran, M. Patient adherence and health management issues: A case study on the effect of medication reminder box. International Journal of Healthcare Management 2020;13(S1):206-211. DOI: 10.1080/20479700.2018.1498200. **Wrong patient population**
62. Janjua, S; Banchoff, E; Threapleton, CJD; Prigmore, S; Fletcher, J; Disler, RT. Digital interventions for the management of chronic obstructive pulmonary disease. Cochrane Database of Systematic Reviews 2021;(4): John Wiley & Sons, Ltd 2021. DOI: 10.1002/14651858.CD013246.pub2. **Wrong intervention**
63. Jensen, L.; Troster, S.M.; Cai, K.; Shack, A.; Chang, Y.-J.R.; Wang, D.; Kim, J.S.; Turial, D.; Bierman, A.S. Improving Heart Failure Outcomes in Ambulatory and Community Care: A Scoping Study. Medical Care Research and Review 2017;74(5):551-581. DOI: 10.1177/1077558716655451. **Wrong study design**
64. Jiang, C.; Zhang, H.; Liu, K.; Zheng, S.; Yang, Y.; Tian, F.; Feng, C. A mobile health solution for medication adherence intervention and its real world evidence. 2018 IEEE 20th International Conference on e-Health Networking, Applications and Services (Healthcom). DOI: 10.1109/HealthCom.2018.8531179. **Wrong intervention**
65. Kannure, M; Hegde, A; Khungar-Pathni, A; Sharma, B; Scuteri, A; Neupane, D; Gandhi, RK; Patel, H; Surendran, S; Jondhale, V; Gupta, S; Phalake, A; Walkar, V; George, R; Mcguire, H; Jain, N; Vijayan, S. Phone calls for improving blood pressure control among hypertensive patients attending private medical practitioners in India: Findings from Mumbai hypertension project. Journal of Clinical Hypertension 2021;23(4):730-737. DOI: 10.1111/jch.14221. **Wrong intervention**
66. Khalil, H; Bell, B; Chambers, H; Sheikh, A; Avery, AJ. Professional, structural and organisational interventions in primary care for reducing medication errors. Cochrane Database of Systematic Reviews 2017;(10): John Wiley & Sons, Ltd 2017. DOI: 10.1002/14651858.CD003942.pub3. **Wrong intervention**
67. Kim, B.Y.B.; Lee, J. Smart devices for older adults managing chronic disease: A scoping review. JMIR mHealth and uHealth 2017;5(5). DOI: 10.2196/mhealth.7141. **Wrong study design**
68. Kim, E.-Y. Development and application of direct data capture for monitoring medication compliance in clinical trials. Healthcare Informatics Research 2017;23(4):249-254. DOI: 10.4258/hir.2017.23.4.249. **Wrong outcomes**
69. Kim, JY; Wineinger, NE; Steinhubl, SR. The Influence of Wireless Self-Monitoring Program on the Relationship Between Patient Activation and Health Behaviors, Medication Adherence, and Blood Pressure Levels in Hypertensive Patients: a Substudy of a Randomized Controlled Trial. J Med Internet Res 2016;18(6):e116. DOI: 10.2196/jmir.5429. **Wrong intervention**
70. Kjos AL; Vaughan AG; Bhargava A. Impact of a mobile app on medication adherence and adherence-related beliefs in patients with type 2 diabetes. J Am Pharm Assoc 2019; 59(2S):S44-S51.e3. DOI: 10.1016/j.japh.2018.12.012. **Wrong outcomes**
71. Konnyu KJ, Yogasingam S, Lépine J, Sullivan K, Alabousi M, Edwards A, Hillmer M, Karunananthan S, Lavis JN, Linklater S, et al. Quality improvement strategies for diabetes care: Effects on outcomes for adults living with diabetes. Cochrane Database of Systematic Reviews 2023;(5). DOI: 10.1002/14651858.CD014513. **Wrong intervention**
72. Kothari, M.; Maidment, I.; Dey, P. Potential advantages and drawbacks to electronic medicine management systems for UK long-term care facilities: a literature review. International Association for Management of Technology IAMOT 2018 Conference Proceedings. **Wrong setting**
73. Kovach, C.R.; Ellis, J.L.; Schiffman, R.F. Is assisted self-management a viable concept for individuals with cognitive impairment? Research in Gerontological Nursing 2018;11(3):115-117. DOI: 10.3928/19404921-20180413-01. **Wrong study design**
74. Kravitz RL; Schmid CH; Marois M; Wilsey B; Ward D; Hays RD; Duan N; Wang Y; MacDonald S; Jerant A; Servadio JL; Haddad D; Sim I. Effect of Mobile Device-Supported Single-Patient Multi-crossover Trials on Treatment of Chronic Musculoskeletal Pain: A Randomized Clinical Trial. JAMA Intern Med Oct 2018;178(10):1368-1377. DOI: 10.1001/jamainternmed.2018.3981. **Wrong intervention**
75. Krick, T.; Huter, K.; Domhoff, D.; Schmidt, A.; Rothgang, H.; Wolf-Ostermann, K. Digital technology and nursing care: A scoping review on acceptance, effectiveness and efficiency studies of informal and formal care technologies. BMC Health Services Research 2019;19(1). DOI: 10.1186/s12913-019-4238-3. **Wrong intervention**
76. Kruse CS, Mileski M, Heinemann K, Huynh H, Leafblad A, Moreno E. Analyzing the Effectiveness of mHealth to Manage Diabetes Mellitus Among Adults Over 50: A Systematic Literature Review. Journal of Multidisciplinary Healthcare 2023;16():101-117. DOI: 10.2147/JMDH.S392693. **Wrong intervention**
77. Kröger, E.; Tatar, O.; Vedel, I.; Giguère, A.M.C.; Voyer, P.; Guillaumie, L.; Grégoire, J.-P.; Guénette, L. Improving medication adherence among community-dwelling seniors with cognitive impairment: a systematic review of interventions. International Journal of Clinical Pharmacy 2017;39(4):641-656. DOI: 10.1007/s11096-017-0487-6. **Wrong outcomes**
78. Lancioni, G.; Singh, N.; O’Reilly, M.; Sigafoos, J.; D’Amico, F.; Pinto, K.; De Vanna, F.; Caffò, A. A technology-aided program for helping persons with Alzheimer’s disease perform daily activities. Journal of Enabling Technologies 2017;11(3):85-91. DOI: 10.1108/JET-03-2017-0011. **Wrong intervention**
79. Lau, Y; Chee, DGH; Chow, XP; Cheng, LJ; Wong, SN. Personalised eHealth interventions in adults with overweight and obesity: A systematic review and meta-analysis of randomised controlled trials. Preventive Medicine 2020; 132. DOI: 10.1016/j.ypmed.2020.106001. **Wrong patient population**
80. Lazaridis, C; Mouselimis, D; Bakogiannis, C; Tsarouchas, A; Antoniadis, A; Papadopoulos, CE; Tzikas, S; Fragakis, N; Vassilikos, VP. The role of the novel MyAlgos e-medicine Platform in promoting patient-centered selfcare management in patients with atrial fibrillation: the emPOWERD trial. European Journal of Preventive Cardiology 2021, 28. Suppl 1. DOI: 10.1093/eurjpc/zwab061.432. **Wrong study design**
81. Lee, K.; Kwon, H.; Lee, B.; Lee, G.; Lee, J.H.; Park, Y.R.; Shin, S.-Y. Effect of self-monitoring on long-term patient engagement with mobile health applications. PLoS ONE 2018;13(7). DOI: 10.1371/journal.pone.0201166. **Wrong intervention**
82. Leon, N; Namadingo, H; Cooper, S; Bobrow, K; Mwantisi, C; Nyasulu, M; Sicwebu, N; Crampin, A; Levitt, N; Farmer, A. Process evaluation of a brief messaging intervention to improve diabetes treatment adherence in sub-Saharan Africa. BMC Public Health (2021) 21(1):1576. DOI: 10.1186/s12889-021-11552-8. **Wrong outcomes**
83. Li, J; Sun, L; Hou, Y; Chen, L. Cost-Effectiveness Analysis of a Mobile-Based Intervention for Patients with Type 2 Diabetes Mellitus. Hindawi International Journal of Endocrinology Volume 2021, Article ID 8827629, 7 pages. https://doi.org/10.1155/2021/8827629. **Wrong intervention**
84. Liu, N.; Yin, J.; Tan, S.S.-L.; Ngiam, K.Y.; Teo, H.H. Mobile health applications for older adults: A systematic review of interface and persuasive feature design. Journal of the American Medical Informatics Association 2021;28(11):2483-2501. DOI: 10.1093/jamia/ocab151. **Wrong intervention**
85. Maddison R; Jiang Y; Stewart R; Scott T; Kerr A; Whittaker R; Benatar J; Rolleston A; Estabrooks P; Dale L. An Intervention to Improve Medication Adherence in People With Heart Disease (Text4HeartII): Randomized Controlled Trial. JMIR Mhealth Uhealth Jun 2021;9(6):e24952. DOI: 10.2196/24952. **Wrong intervention**
86. Majumder, S.; Aghayi, E.; Noferesti, M.; Memarzadeh-Tehran, H.; Mondal, T.; Pang, Z.; Deen, M.J. Smart homes for elderly healthcare—Recent advances and research challenges. Sensors (Switzerland) 2017;17(11). DOI: 10.3390/s17112496. **Wrong study design**
87. Mason, M.; Cho, Y.; Rayo, J.; Gong, Y.; Harris, M.; Jiang, Y. Technologies for Medication Adherence Monitoring and Technology Assessment Criteria: Narrative Review. JMIR mHealth and uHealth 2022;10(3). DOI: 10.2196/35157. **Wrong outcomes**
88. McBride CM; Morrissey EC; Molloy GJ. Patients' Experiences of Using Smartphone Apps to Support Self-Management and Improve Medication Adherence in Hypertension: Qualitative Study. JMIR Mhealth Uhealth Oct 2020;8(10):e17470. DOI: 10.2196/17470. **Wrong comparator**
89. Mackridge AJ, Wood EM, Hughes DA. Improving medication adherence in the community: a purposive umbrella review of effective patient-directed interventions that are readily implementable in the United Kingdom National Health Service. International Journal of Clinical Pharmacy 2025;47(3):640-653. DOI: 10.1007/s11096-025-01885-4. **Wrong outcomes**
90. McGrattan M; Ryan C; Barry HE; Hughes CM. Interventions to Improve Medicines Management for People with Dementia: A Systematic Review. Drugs Aging Dec 2017;34(12):907-916. DOI: 10.1007/s40266-017-0505-3. **Wrong intervention**
91. Milewski K; Małecki A; Orszulik-Baron D; Kachel M; Hirnle P; Orczyk M; Dunal R; Mikołajowski G; Janas A; Nowak Z; Kozak K; Roskiewicz W; Nierwińska K; Izworski A; Rybicki A; Buszman PP; Piotrowicz R; Buszman PE. The use of modern telemedicine technologies in an innovative optimal cardiac rehabilitation program for patients after myocardial revascularization: Concept and design of RESTORE, a randomized clinical trial. Cardiol J 2019;26(5):594-603. DOI: 10.5603/CJ.a2018.0157. **Wrong intervention**
92. Morawski, K; Ghazinouri, R; Krumme, A; McDonough, J; Durfee, E; Oley, L; Mohta, N; Juusola, J; Choudhry, NK. Rationale and design of the Medication adherence Improvement Support App For Engagement-Blood Pressure (MedISAFE-BP) trial. American Heart Journal 2017; 186:40‐47. DOI: 10.1016/j.ahj.2016.11.007. **Wrong study design**
93. Morrissey, EC; Casey, M; Glynn, LG; Walsh, JC; Molloy, GJ. Smartphone apps for improving medication adherence in hypertension: patients' perspectives. Patient Preference and Adherence 2018; 12:813-822. DOI: 10.2147/PPA.S145647. **Wrong comparator**
94. Mosnaim GS; Stempel DA; Gonzalez C; Adams B; BenIsrael-Olive N; Gondalia R; Kaye L; Shalowitz M; Szefler S. The Impact of Patient Self-Monitoring Via Electronic Medication Monitor and Mobile App Plus Remote Clinician Feedback on Adherence to Inhaled Corticosteroids: A Randomized Controlled Trial. J Allergy Clin Immunol Pract Apr 2021; 9(4):1586-1594. DOI: 10.1016/j.jaip.2020.10.064. **Wrong patient population**
95. Nelson LA; Mulvaney SA; Johnson KB; Osborn CY. mHealth Intervention Elements and User Characteristics Determine Utility: A Mixed-Methods Analysis. Diabetes Technol Ther Jan 2017;19(1):9-17. DOI: 10.1089/dia.2016.0294. **Wrong comparator**
96. Newman-Casey, P.A.; Niziol, L.M.; Lee, P.P.; Musch, D.C.; Resnicow, K.; Heisler, M. The Impact of the Support, Educate, Empower Personalized Glaucoma Coaching Pilot Study on Glaucoma Medication Adherence. Ophthalmology Glaucoma 2020;3(4):228-237. DOI: 10.1016/j.ogla.2020.04.013. **Wrong outcomes**
97. Newman-Casey, P.A.; Niziol, L.M.; Mackenzie, C.K.; Resnicow, K.; Lee, P.P.; Musch, D.C.; Heisler, M. Personalized behavior change program for glaucoma patients with poor adherence: A pilot interventional cohort study with a pre-post design. Pilot and Feasibility Studies 2018;4(1). DOI: 10.1186/s40814-018-0320-6. **Wrong study design**
98. Nilsson, M.Y.; Andersson, S.; Magnusson, L.; Hanson, E. Ambient assisted living technology-mediated interventions for older people and their informal carers in the context of healthy ageing: A scoping review. Health Science Reports 2021;4(1). DOI: 10.1002/hsr2.225. **Wrong study design**
99. O'Brien, K; Light, SW; Bradley, S; Lindquist, L. Optimizing voice-controlled intelligent personal assistants for use by home-bound older adults. J Am Geriatr Soc. 2022;70:1504–1509. DOI: 10.1111/jgs.17625. **Wrong intervention**
100. Oh SW; Kim KK; Kim SS; Park SK; Park S. Effect of an Integrative Mobile Health Intervention in Patients With Hypertension and Diabetes: Crossover Study. JMIR Mhealth Uhealth 2022;10(1):e27192. DOI: 10.2196/27192. **Wrong intervention**
101. Olamoyegun MA; Emuoyibofarhe OJ; Ala OA; Ugwu E. Mobile Phone Use in the Management of Diabetes in Nigeria: A New Potential Weapon. West Afr J Med Jul-Aug 2020;37(3):201-208. **Wrong study design**
102. Palmer, MJ; Machiyama, K; Woodd, S; Gubijev, A; Barnard, S; Russell, S; Perel, P; Free, C. Mobile phone‐based interventions for improving adherence to medication prescribed for the primary prevention of cardiovascular disease in adults. Cochrane Database of Systematic Reviews 2021;3: John Wiley & Sons, Ltd 2021. DOI: 10.1002/14651858.CD012675.pub3. **Wrong intervention**
103. Pandey, A.; Krumme, A.A.; Patel, T.; Choudhry, N.K. The impact of text messaging on medication adherence and exercise among postmyocardial infarction patients: Randomized controlled pilot trial. JMIR mHealth and uHealth 2017;5(8). DOI: 10.2196/mhealth.7144. **Wrong outcomes**
104. Park DY; Goering EM; Head KJ; Bartlett Ellis RJ. Implications for Training on Smartphone Medication Reminder App Use by Adults With Chronic Conditions: Pilot Study Applying the Technology Acceptance Model. JMIR Form Res Nov 2017;1(1):e5. DOI: 10.2196/formative.8027. **Wrong intervention**
105. Pereira, L.; Dias, A.; Queirós, A.; Rocha, N.P. Technologies for ageing in place: A systematic review of reviews and meta-analyses. Peixeto et al. (Eds.) BIOSTEC 2017, CCIS 881, 331-353, 2018. DOI: 10.1007/978-3-319-94806-5_18. **Wrong intervention**
106. Pérez-Jover, V.; Mira, J.J.; Carratala-Munuera, C.; Gil-Guillen, V.F.; Basora, J.; López-Pineda, A.; Orozco-Beltrán, D. Inappropriate use of medication by elderly, polymedicated, or multipathological patients with chronic diseases. International Journal of Environmental Research and Public Health 2018;15(2). DOI: 10.3390/ijerph15020310. **Wrong outcomes**
107. Pérez-Jover, V.; Sala-González, M.; Guilabert, M.; Mira, J.J. Mobile apps for increasing treatment adherence: Systematic review. Journal of Medical Internet Research 2019;21(6). DOI: 10.2196/12505. **Wrong outcomes**
108. Petrie, H.; Darzentas, J.S. Digital technology for older people: A review of recent research. The Oxford Handbook of Digital Technology and Society 2020; 136-185. DOI: 10.1093/oxfordhb/9780190932596.013.6. **Wrong study design**
109. Pinho, S.; Cruz, M.; Ferreira, F.; Ramalho, A.; Sampaio, R. Improving medication adherence in hypertensive patients: A scoping review. Preventive Medicine 2021;146. DOI: 10.1016/j.ypmed.2021.106467. **Wrong study design**
110. Polenick, CA., Stanz, SD., Leggett, AN., Maust, DT., Hodgson, NA., Kales, HC. Stressors and Resources Related to Medication Management: Associations With Spousal Caregivers’ Role Overload. Gerontologist, 2020, Vol. 60, No. 1, 165–173 doi:10.1093/geront/gny130 **Wrong intervention**
111. Prayaga, R.B.; Jeong, E.W.; Feger, E.; Noble, H.K.; Kmiec, M.; Prayaga, R.S. Improving refill adherence in medicare patients with tailored and interactive mobile text messaging: Pilot study. JMIR mHealth and uHealth 2018;6(1). DOI: 10.2196/mhealth.8930. **Wrong outcomes**
112. Redfern J et al. A digital health intervention for cardiovascular disease management in primary care (CONNECT) randomized controlled trialDigital Medicine (2020) 3:117. https://doi.org/10.1038/s41746-020-00325-z. **Wrong intervention**
113. Rice DR; Kaplan TB; Hotan GC; Vogel AC; Matiello M; Gillani RL; Hutto SK; Ham AS; Klawiter EC; George IC; Galetta K; Mateen FJ. Electronic pill bottles to monitor and promote medication adherence for people with multiple sclerosis: A randomized, virtual clinical trial. J Neurol Sci Sep 2021;428():117612. DOI: 10.1016/j.jns.2021.117612. **Wrong outcomes**
114. Rief, JJ; Hamm, ME; Zickmund, SL; Nikolajski, C; Lesky, D; Hess, R; Fischer, GS; Weimer, M; Clark, S; Zieth, C; et al. Using Health Information Technology to Foster Engagement: patients' Experiences with an Active Patient Health Record. Health Communication, 32:3, 310-319, DOI: 10.1080/10410236.2016.1138378. **Wrong intervention**
115. Rocha, N.P.; Santos, M.; Cerqueira, M.; Queirós, A.Mobile health to support ageing in place: A systematic review of reviews and meta-analyses. International Journal of E-Health and Medical Communications 2019;10(3):1-21. DOI: 10.4018/IJEHMC.2019070101. **Wrong intervention**
116. Rodríguez, M.D.; Beltrán, J.; Valenzuela-Beltrán, M.; Cruz-Sandoval, D.; Favela, J. Assisting older adults with medication reminders through an audio-based activity recognition system. Personal and Ubiquitous Computing 2021;25(2):337-351. DOI: 10.1007/s00779-020-01420-4. **Wrong outcomes**
117. Ross, EL; Jamison, RN; Nicholls, L; Perry, BM; Nolen, KD. Clinical Integration of a Smartphone App for Patients With Chronic Pain: Retrospective Analysis of Predictors of Benefits and Patient Engagement Between Clinic Visits. Journal of Medical Internet Research 2020;22(4). DOI:10.2196/16939. **Wrong intervention**
118. Ruksakulpiwat, S. Intervention Enhancing Medication Adherence in Stroke Patients: An Integrative Review. Siriraj Medical Journal 2021;73(7):429-444. DOI: 10.33192/SMJ.2021.57. **Wrong study design**
119. Saha, S.K.; Adhikary, A.; Jha, A.; Mehta, V.K. Use of interventions to overcome medication non-adherence. International Journal of Asian Business and Information Management 2021;12(3):289-318. DOI: 10.4018/IJABIM.20210701.oa18. **Wrong outcomes**
120. Sanaei H. The effect of using disease management Smartphone based application on perceived stress and self-efficacy of caregivers of the elderly with Parkinson's disease. Clinical Trial Protocol, Iranian Registry of Clinical Trials 2020. **Wrong study design**
121. Schorr, E.N.; Gepner, A.D.; Dolansky, M.A.; Forman, D.E.; Park, L.G.; Petersen, K.S.; Still, C.H.; Wang, T.Y.; Wenger, N.K. Harnessing Mobile Health Technology for Secondary Cardiovascular Disease Prevention in Older Adults: A Scientific Statement from the American Heart Association Circulation: Cardiovascular Quality and Outcomes 2021; 659-676. DOI: 10.1161/HCQ.0000000000000103. **Wrong study design**
122. Sefcik JS; Johnson MJ; Yim M; Lau T; Vivio N; Mucchiani C; Cacchione PZ. Stakeholders' Perceptions Sought to Inform the Development of a Low-Cost Mobile Robot for Older Adults: A Qualitative Descriptive Study. Clin Nurs Res Feb 2018;27(1):61-80. DOI: 10.1177/1054773817730517. **Wrong outcomes**
123. Senoo K; Miki T; Ohkura T; Iwakoshi H; Nishimura T; Shiraishi H; Teramukai S; Matoba S. A Smartphone App to Improve Oral Anticoagulation Adherence in Patients With Atrial Fibrillation: Prospective Observational Study. JMIR Mhealth Uhealth Jan 2022;10(1):e30807. DOI: 10.2196/30807. **Wrong outcomes**
124. Shade, MY; Rector, K; Soumana, R; Kupzyk, K. Voice Assistant Reminders for Pain Self-Management Tasks in Aging Adults. Journal of Gerontological Nursing 2020;46(10):27-33. DOI: 10.3928/00989134-20200820-03. **Wrong comparator**
125. Shetty KD; Chen AY; Rose AJ; Liu HH. Effect of the ExactCare medication care management model on adherence, health care utilization, and costs. J Manag Care Spec Pharm May 2021;27(5):574-585. DOI: 10.18553/jmcp.2021.20431. **Wrong intervention**
126. Shishehgar, M.; Kerr, D.; Blake, J. A systematic review of research into how robotic technology can help older people. Smart Health 2018;7-8():1-18. DOI: 10.1016/j.smhl.2018.03.002. **Wrong outcomes**
127. Sikorskii A; Given CW; Given BA; Banik A; Krauss JC. Patient Engagement With an Automated Telephone Symptom Management Intervention: Predictors and Outcomes. Ann Behav Med Jun 2020;54(7):484-494. DOI: 10.1093/abm/kaz067. **Wrong intervention**
128. Simon, S.T.; Kini, V.; Levy, A.E.; Ho, P.M. Medication adherence in cardiovascular medicine The BMJ 2021;374. DOI: 10.1136/bmj.n1493. **Wrong study design**
129. Singh, N.; Varshney, U. IT-based reminders for medication adherence: systematic review, taxonomy, framework and research directions. European Journal of Information Systems 2020;29(1):84-108. DOI: 10.1080/0960085X.2019.1701956. **Wrong outcomes**
130. Skrabal Ross X; Gunn KM; Patterson P; Olver I. Mobile-Based Oral Chemotherapy Adherence-Enhancing Interventions: Scoping Review. JMIR Mhealth Uhealth Dec 2018;6(12):e11724. DOI: 10.2196/11724. **Wrong study design**
131. Smith, D.; Lovell, J.; Weller, C.; Kennedy, B.; Winbolt, M.; Young, C.; Ibrahim, J. A systematic review of medication nonadherence in persons with dementia or cognitive impairment. PLoS ONE 2017;12(2). DOI: 10.1371/journal.pone.0170651. **Wrong outcomes**
132. Smith, SM; Wallace, E; O'Dowd, T; Fortin, M. Interventions for improving outcomes in patients with multimorbidity in primary care and community settings. Cochrane Database of Systematic Reviews 2021(1), John Wiley & Sons, Ltd 2021. DOI: 10.1002/14651858.CD006560.pub4. **Wrong intervention**
133. Sohn A; Speier W; Lan E; Aoki K; Fonarow GC; Ong MK; Arnold CW. Integrating remote monitoring into heart failure patients' care regimen: A pilot study. PLoS One 2020;15(11):e0242210. DOI: 10.1371/journal.pone.0242210. **Wrong intervention**
134. Sokullu, R.; Akkaş, M.A.; Demir, E. IoT supported smart home for the elderly. Internet of Things (Netherlands) 2020;11. DOI: 10.1016/j.iot.2020.100239. **Wrong study design**
135. Stara V; Vera B; Bolliger D; Paolini S; de Jong M; Felici E; Koenderink S; Rossi L; Von Doellen V; di Rosa M. Toward the Integration of Technology-Based Interventions in the Care Pathway for People with Dementia: A Cross-National Study. Int J Environ Res Public Health Oct 2021;18(19). DOI: 10.3390/ijerph181910405. **Wrong intervention**
136. Steinert A; Eicher C; Haesner M; Steinhagen-Thiessen E. Effects of a long-term smartphone-based self-monitoring intervention in patients with lipid metabolism disorders. Assist Technol 2020;32(2):109-116. DOI: 10.1080/10400435.2018.1493710. **Wrong outcomes**
137. Steinert, A.; Haesner, M.; Steinhagen-Thiessen, E. App-based self-monitoring in type 2 diabetes. Z Gerontol Geriat 2017;50(6):516–523. DOI 10.1007/s00391-016-1082-5. **Wrong intervention**
138. Sua, Y.S.; Jiang, Y.; Thompson, D.R.; Wang, W. Effectiveness of mobile phone-based self-management interventions for medication adherence and change in blood pressure in patients with coronary heart disease: A systematic review and meta-analysis. European Journal of Cardiovascular Nursing 2020;19(3):192-200. DOI: 10.1177/1474515119895678. **Wrong intervention**
139. Sumner, J.; Chong, L.S.; Bundele, A.; Wei Lim, Y. Co-Designing Technology for Aging in Place: A Systematic Review. Gerontologist 2021;61(7):E395-E409. DOI: 10.1093/geront/gnaa064. **Wrong intervention**
140. Suzuki R; Hasegawa T. Evaluation of a one-dose package medication support system for community-based elderly patients and eldercare facilities. Am J Health Syst Pharm May 2018;75(9):e202-e212. DOI: 10.2146/ajhp170176. **Wrong comparator**
141. Tian, XY; Yu, MC; Sun, YF; Yan, HY; Ma, HQ; Jiang, L; Zhu, YQ; Wang, LF; Ding, Q; Liu, AX. Evidence-based summary for the safety of multiple medication in elderly patients with ischemic stroke. Annals of Translational Medicine 2022;10(4). DOI: 10.21037/atm-22-453. **Wrong outcomes**
142. Tumuhimbise, W.; Musiimenta, A. A review of mobile health interventions for public private mix in tuberculosis care. Internet Interventions 2021;25. DOI: 10.1016/j.invent.2021.100417. **Wrong outcomes**
143. Turjamaa, R.; Kapanen, S.; Kangasniemi, M. How smart medication systems are used to support older people's drug regimens: A systematic literature review. Geriatric Nursing 2020;41(6):677-684. DOI: 10.1016/j.gerinurse.2020.02.005. **Wrong outcomes**
144. van de Hei, SJ; Dierick, BJH; Aarts, JEP; Kocks, JWH; van Boven, JFM. Personalized Medication Adherence Management in Asthma and Chronic Obstructive Pulmonary Disease: A Review of Effective Interventions and Development of a Practical Adherence Toolkit. Journal of Allergy and Clinical Immunology-In Practice 2021;9(11):3979-3994. DOI: 10.1016/j.jaip.2021.05.025. **Wrong study design**
145. Van der Roest, HG; Wenborn, J; Pastink, C; Dröes, RM; Orrell, M. Assistive technology for memory support in dementia. Cochrane Database of Systematic Reviews 2017;6: John Wiley & Sons, Ltd 2017. DOI: 10.1002/14651858.CD009627.pub2. **Wrong intervention**
146. Van Grootven B; van Achterberg T. The European Union's Ambient and Assisted Living Joint Programme: An evaluation of its impact on population health and well-being. Health Informatics J Mar 2019;25(1):27-40. DOI: 10.1177/1460458216683535. **Wrong study design**
147. Volpp KG; Troxel AB; Mehta SJ; Norton L; Zhu J; Lim R; Wang W; Marcus N; Terwiesch C; Caldarella K; Levin T; Relish M; Negin N; Smith-McLallen A; Snyder R; Spettell CM; Drachman B; Kolansky D; Asch DA. Effect of Electronic Reminders, Financial Incentives, and Social Support on Outcomes After Myocardial Infarction: The HeartStrong Randomized Clinical Trial. JAMA Intern Med Aug 2017;177(8):1093-1101. DOI: 10.1001/jamainternmed.2017.2449. **Wrong intervention**
148. Waughtal, J; Luong, P; Sandy, L; Chavez, C; Ho, PM; Bull, S. Nudge me: tailoring text messages for prescription adherence through N-of-1 interviews. Translational Behavioral Medicine 2021;11(10):1832-1838. DOI: 10.1093/tbm/ibab056. **Wrong outcomes**
149. Wei X et al. Protocol for a randomised controlled trial to evaluate the effectiveness of improving tuberculosis patients’ treatment adherence via electronic monitors and an app versus usual care in Tibet. Trials (2019) 20:273. https://doi.org/10.1186/s13063-019-3364-x. **Wrong study design**
150. Welch, V.; Mathew, C.M.; Babelmorad, P.; Li, Y.; Ghogomu, E.T.; Borg, J.; Conde, M.; Kristjansson, E.; Lyddiatt, A.; Marcus, S.; Nickerson, J.W.; Pottie, K.; Rogers, M.; Sadana, R.; Saran, A.; Shea, B.; Sheehy, L.; Sveistrup, H.; Tanuseputro, P.; Thompson-Coon, J.; Walker, P.; Zhang, W.; Howe, T.E. Health, social care and technological interventions to improve functional ability of older adults living at home: An evidence and gap map. Campbell Systematic Reviews 2021;17(3). DOI: 10.1002/cl2.1175. **Wrong study design**
151. Wiecek, E; Torres-Robles, A; Cutler, RL; Benrimoj, SI; Garcia-Cardenas, V. Impact of a Multicomponent Digital Therapeutic Mobile App on Medication Adherence in Patients with Chronic Conditions: Retrospective Analysis. Journal of Medical Internet Research 2020;22(8). DOI: 10.2196/17834. **Wrong outcomes**
152. Wong K. Kardia – A smartphone-based care model for outpatient cardiac rehabilitation. Kardia Study Protocol Version 1.1 dated 31 May 2017. **Wrong study design**
153. Wu Z, Lu L, Li Y, Chen J, Zhang Z, Ning C, Yuan Z, Pan Q, Shen X, Zhang W. Effect of mobile health reminders on tuberculosis treatment outcomes in Shanghai, China: A prospective cohort study. Front Public Health 2023;11():923319. DOI: 10.3389/fpubh.2023.923319. **Wrong patient population**
154. Xiao M; Lei X; Zhang F; Sun Z; Harris VC; Tang X; Yan L. Home Blood Pressure Monitoring by a Mobile-Based Model in Chongqing, China: A Feasibility Study. Int J Environ Res Public Health Sep 2019;16(18). DOI: 10.3390/ijerph16183325. **Wrong intervention**
155. Xu, R.; Xing, M.; Javaherian, K.; Peters, R.; Ross, W.; Bernal-Mizrachi, C. Improving HbA1c with Glucose Self-Monitoring in Diabetic Patients with EpxDiabetes, a Phone Call and Text Message-Based Telemedicine Platform: A Randomized Controlled Trial. Telemedicine and e-Health 2020;26(6):784-793. DOI: 10.1089/tmj.2019.0035. **Wrong patient population**
156. Xu W, Huang X, Lin Q, Wu T, Guan C, Lv M, Hu W, Dai H, Chen P, Li M, Zhang F, Zhang J. Application of Alfalfa App in the management of oral anticoagulation in patients with atrial fibrillation: a multicenter randomized controlled trial. BMC Med Inform Decis Mak Oct 2024;24(1):294. DOI: 10.1186/s12911-024-02701-1. **Wrong intervention**
157. Xu, Y.; Ye, H.; Zhu, Y.; Du, S.; Xu, G.; Wang, Q. The efficacy of mobile health in alleviating risk factors related to the occurrence and development of coronary heart disease: A systematic review and meta-analysis. Clinical Cardiology 2021;44(5):609-619. DOI: 10.1002/clc.23596. **Wrong outcomes**
158. Yasmin F; Nahar N; Banu B; Ali L; Sauerborn R; Souares A. The influence of mobile phone-based health reminders on patient adherence to medications and healthy lifestyle recommendations for effective management of diabetes type 2: a randomized control trial in Dhaka, Bangladesh. BMC Health Serv Res Jun 2020;20(1):520. DOI: 10.1186/s12913-020-05387-z. **Wrong intervention**
159. Yi, J.Y.; Kim, Y.; Cho, Y.-M.; Kim, H. Self-management of chronic conditions using mhealth interventions in Korea: A systematic review. Healthcare Informatics Research 2018;24(3):187-197. DOI: 10.4258/hir.2018.24.3.187. **Wrong intervention**
160. Yuting Z, Xiaodong T, Qun W. Effectiveness of a mHealth intervention on hypertension control in a low-resource rural setting: A randomized clinical trial. Front Public Health 2023;11():1049396. DOI: 10.3389/fpubh.2023.1049396. **Wrong intervention**
161. Zaman, S.B.; Khan, R.K.; Evans, R.G.; Thrift, A.G.; Maddison, R.; Shariful Islam, S.M. Exploring Barriers to and Enablers of the Adoption of Information and Communication Technology for the Care of Older Adults With Chronic Diseases: Scoping Review. JMIR Aging 2022;5(1). DOI: 10.2196/25251. **Wrong intervention**
162. Zamanillo-Campos, R.; Serrano-Ripoll, M.J.; Taltavull-Aparicio, J.M.; Gervilla-García, E.; Ripoll, J.; Fiol-Deroque, M.A.; Boylan, A.-M.; Ricci-Cabello, I. Patients’ Views on the Design of DiabeText, a New mHealth Intervention to Improve Adherence to Oral Antidiabetes Medication in Spain: A Qualitative Study. International Journal of Environmental Research and Public Health 2022;19(3). DOI: 10.3390/ijerph19031902. **Wrong outcomes**
163. Zárate-Bravo E; García-Vázquez JP; Torres-Cervantes E; Ponce G; Andrade ÁG; Valenzuela-Beltrán M; Rodríguez MD. Supporting the Medication Adherence of Older Mexican Adults Through External Cues Provided With Ambient Displays: Feasibility Randomized Controlled Trial. JMIR Mhealth Uhealth Mar 2020;8(3):e14680. DOI: 10.2196/14680. **Wrong outcomes**
164. Zeng, Z.; Wu, T.; Lv, M.; Qian, J.; Chen, M.; Fang, Z.; Jiang, S.; Zhang, J. Impact of mobile health and telehealth technology on medication adherence of stroke patients: a systematic review and meta-analysis of randomized controlled trials. International Journal of Clinical Pharmacy 2022;44(1):4-14. DOI: 10.1007/s11096-021-01351-x. **Wrong outcomes**
165. Zhao, Y.-Y.; Dang, F.-P.; Zhai, T.-T.; Li, H.-J.; Wang, R.-J.; Ren, J.-J. The effect of text message reminders on medication adherence among patients with coronary heart disease: A systematic review and meta-analysis. Medicine (United States) 2019;98(52). DOI: 10.1097/MD.0000000000018353. **Wrong intervention**
166. Zhu Y, Zhao Y, Wu Y. Effectiveness of mobile health applications on clinical outcomes and health behaviors in patients with coronary heart disease: A systematic review and meta-analysis. Int J Nurs Sci. 2024 Mar 10;11(2):258-275. doi: 10.1016/j.ijnss.2024.03.012. **Wrong intervention**

**Appendix Table A3: Reminder original studies**

| Reminder Study | Intervention  Details | Length | Cost | Outcomes  Service use | & Primary  Patient satisfaction and carer burden | Findings  Health | Prof. well-being | Equity | Type of study | MMAT Quality |
| --- | --- | --- | --- | --- | --- | --- | --- | --- | --- | --- |
| Bediang et al. (2018) | SMS with tuberculosis patients  Mean age: NA but age ranges from 18 to 80 years: 20.1% 18-25, 58.4% 26-40, 16.5% 41-55, 5% 56-80 years.  n=279 | 6 months |  |  | Satisfaction for general management of patients (n.s., very high and similar, above 99%, in both groups: ig 99.5 and cg 99.2%, p=0.41) | Cured (in favor of intervention but n.s.) OR at 5 months: 1.45 [0.81, 2.56]; p=0.20), at 6 months OR = 1.06 [0.65, 1.73]; p=0.79 (drop-outs of the cured at last month) |  |  | RCT | 3/5 |
| Buis et al. (2017) | SMS with hypertension patients  Mean age: 49 years  n=123 | 1 month |  |  |  | SBP and DBP improved compared to control (usual care) but n.s.  SBP: ig mean change -12.6, SD 24.0 and cg mean change -11.3, SD 25.5 mmHg, p=0.78; DBP: ig mean change -4.9, SD 13.1 mmHg and cg mean change -3.3, SD 14.3 mmHg, p=0.54) |  |  | RCT | 3/5 |
| Criner et al. (2021) | Medication reminders through the BreatheMate device and smartphone app, cg: no reminders  Mean age: ig 66.6, cg 66.7 years  n=138 | 6 months |  |  |  | The mean baseline total and domain CCQ scores (n.s., favoring control group) were higher (worse) for the control group compared with the intervention group indicating worse baseline COPD symptom, functional, and mental status. |  |  | RCT | 1/5 |
| Dıgın et al. (2022) | SMS with cataract surgery patients  Mean age: ig 57.2, cg 58 years  n=82 | 7 days |  |  |  | SAI-score (anxiety) change (n.s.), favoring control (routine care), p=0.079, Effect size r=0.193.  Mean difference: Intervention +24.9 SD(9.2), Control +21.5 SD(10.5).  The SMS group that started with lower SAI mean scores than the control group had lower scores (p=0.001) on the seventh postoperative day also. |  |  | RCT | 3/5 |
| Farmer et al. (2021) | Mobile app with type 2 diabetes patients  Mean age: ig 56.8, cg 57.4 years  n=1186 | 12 months |  |  | Satisfaction with health care (n.s.), change from baseline: ig +0.27, SD(0.74), cg +0.21, SD(0.74); Mean difference −0.030  (−0.038 to 0.099), p=0.385 | Several mean difference of change from baseline to 12 months results favored intervention; SBP: −3.46 mmHg, CI(−5.44 to −1.48) p=0.001 (s.), 10-year risk of coronary heart disease: −0.71% CI(−1.46 to 0.04, p=0.064) (n.s.), proportion of participants meeting treatment goals: ig 36.0% vs. cg 26.8% (Relative risk 1.36 (1.13 to 1.63, p=0.001) (s.), HbA1c: The change from baseline to 1 year was: ig −1.15% SD(2.81) [−12.53 (30.72) mmol/mol] and cg: −1.19 SD(2.86) [−13.02 (31.27) mmol/mol]. The overall adjusted difference in HbA1c (95% CI) between groups was −0.08% (−0.31 to 0.16) [−0.82 (−3.44 to 1.79), p=0.537] (n.s., also treatment more effective with less than 7 years of diabetes, p=0.05, and those older than 55 years, n.s.), total/HDL cholesterol: ig −0.03 SD(1.3), cg 0.01 SD(1.1), mean difference −0.03 CI(− 0.17 to 0.10) p=0.633 (n.s.), health status EQ-5D ig 0.009 SD(0.160), cg −0.004 SD(0.157), mean difference −0.011 (−0.007 to 0.030) p=0.202 (n.s.), BMI: ig 0.03 SD(2.14), cg −0.07 SD(3.76), Mean difference −0.08 CI (-0.45 to 0.29) p-value 0.669 (n.s.) |  |  | RCT | 5/5 |
| Graetz et al. (2024) | Smart pill bottle with SMS reminders (ig) for missed or incorrect doses, and bidirectional text messaging to monitor patient-reported outcomes vs. usual care while using the smart pill bottle (cg: no reminders).  Mean age: 59.2 years  n=28 | 90 days |  |  |  | 12.5% of ig participants had reductions in GMH (global mental health score) compared with 69.2% of cg (p=0.011).  10% of ig participants reported more severe symptoms at follow-up relative to enrollment compared with 57.1% of cg participants (p=0.019).  (Both are s., favoring intervention.)  87.5% of ig participants had reductions in GPH (global physical health score) compared with 66.7% of cg (p=0.292) (n.s., favoring control). |  |  | RCT | 3/5 |
| Greer et al. (2020) | Mobile app with cancer patients and oral therapy  Mean age: ig 52.85, cg 53.76 years  n=181 | 12 weeks |  | Emergency depart-ment (ED) visits: standard care cg 0.14 SE(0.04), mobile app ig 0.16 SE(0.04); mean difference  -0.03 SE(0.06); Hospitaliza-tions: cg 0.15 SE(0.07) ig 0.20 SE(0.07), mean difference -0.05, SE(0.10), 95% CI(-0.24 to 0.15) p=0.640, (both ns. favoring treatment) | Treatment satisfaction (FACIT-TS-PS): Clinician expla-nations -0.09 (0.23), Inter-personal treat-ment -0.16 (0.15), Com-prehensive care -0.40 (0.62), Nursing communica-tion +0.39 (0.26), Trust and confi-dence in clini-cians +0.02 (0.16) (all n.s., both groups are weaker post-treat-ment. Trust and confi-dence in clini-cians p=0.898 and Nursing communica-tion p=0.138 weakened more in the app group, all others favor treatment) | Symptoms (favoring control): severity: +0.04 (0.19), interference +0.05 (0.30); QoL (favoring intervention) -2.28 (1.62),  (all n.s. results, noting that the intervention may help people with certain risk factors, such as difficulties with adherence or elevated anxiety, as related adherence results were statistically significant.) |  |  | RCT | 3/5 |
| Habib et al. (2021) | Mobile app with patients discharged from the hospital  Mean age: 64.6 years  n=66 | 1 month |  | Rehospitalizations are lower in the app group (8.7%) compared to control (15.4%), ED visits contrarily (21.7% versus 19.2%). |  |  |  |  | RCT | 3/5 |
| Huang et al. (2019) | Mobile app with type 2 diabetes patients  Mean age: NA but median (range): ig 51.5 (22-69), cg 52 (28-67) years  n=51 | 12 weeks |  |  |  | Hemoglobin A1c (%): Adjusted mean difference (95% CI) -0.42 (-1.89 to 1.06) p=0.57; LDL (mmol/L) +0.11 (-0.20 to 0.06) p=0.75; HDL: -0.09 (-0.56 to 0.77) p=0.14; Total cholesterol -0.02 (–0.69 to 0.72) p=0.052; BMI +0.02 (-1.13, 1.10) p=0.98. (all n.s.) |  |  | RCT | 3/5 |
| Li et al. (2021) | Mobile app with poly-pharmacy patients  Mean age: ig 58.92, cg 60.04 years  n=124 | 12 months |  |  |  | The Probability of HbA1c ≤6.5% (s. at months 9 and 12), month 9 difference in probability +0.30; 95%CI 0.03 to 0.48; p=0.027) and month 12 difference in probability +0.22; 95%CI 0.01 to 0.44; p=0.039; the probability of total cholesterol ≤5.2mmol/L (s. at month 3, difference in probability 0.16; 95%CI,−0.01 to −0.30; p=0.031); LDL-C level was lower (s.) in the Perx group at month 3, difference in level −0.58; 95%CI −1.07 to −0.09; p=0.019; FBG, NFBG, creatinine, TFT, systolic and diastolic blood pressure, weight or percentage weight change (all n.s., blood glucose and pressure, and weigh decreasing direction with intervention); (Basing on adherence tests only, reports that treatment more effective for obesity, taking medications for diabetes and taking ≤4 medications.) |  |  | RCT | 3/5 |
| Marvel et al. (2021) | Mobile app, smartwatch, BP-monitor with patients discharged from hospital  Mean age: ig 59.2, cg 65.4  n=1064 | 1 month |  | Hospitali-zation 30-days post-discharge risk was 52% lower in the DHI group as compared with the control group (HR, 0.48 [95% CI 0.26 to 0.88]; p=0.018). ED visits that did not result in re-admission: no difference in hazard (HR, 1.45 [95% CI, 0.69–2.98], p=0.33) (n.s.); Follow-up visits and CR not compared to control group. |  |  |  |  | NRCT | 4/5 |
| Mauro et al. (2019) | Smart pill bottle and text message reminders with multiple myeloma patients new to lenalidomide therapy, compared to control (the device alerts deactivated and no text messages)  Mean age: 77.25 years  n=40 | 6 months | Cost-ef-fective-ness:  Annual cost of USD 1210 per patient (with 1 pharma-cist inter-vention and median adher-ence im-prove-ment of 12.6 per-centage points) resulted in $96.03 ICER per one per-centage  point adher-ence increase |  |  |  |  |  | RCT | 2/5 |
| McGillicuddy et al. (2020) | Mobile app reminders with kidney transplant recipients using medication trays, cg: no app reminders and no tray alerts.  Mean age: ig 52.1, cg 51.5  n=82 | 12 months |  |  |  | Tacrolimus coefficient of variation (CV) (Mean/SD×100%) (s.): reduction in mean tacrolimus  CVs (p=0.046), the proportion of tacrolimus CV<40% (p= 0.001), compared to the control. |  |  | RCT | 3/5 |
| Morawski et al. (2018) | Mobile app with hypertension patients  Mean age: ig 51.7, cg 52.4  n=412 | 12 weeks |  |  |  | Mean SBP (ns): Absolute difference -0.1 CI(-3.2 to 3.1), p=0.97, BP <140/90 mm Hg (ns): OR=0.8, (95% CI(0.5 to 1.3), p=0.34; SBP differences between intervention and control groups (95% CI) from interaction terms for subgroups, all are ns. Sex and group (ig) interaction term p=0.47, Female: +1.53 mmHg (−2.46 to 5.51), Male: −0.91 (−6.29 to 4.47); Age and ig interaction term p=0.31, Age at or below median: −1.32 mmHg (−5.83 to 3.20), above median: +2.02 (−2.60 to 6.64); Baseline SBP and ig interaction term p=0.17, Baseline SBP<160 mmHg: −0.48 mmHg (−3.92 to 2.97), ≥160 mmHg: +5.31 (−2.92 to 13.54); Adherence and ig interaction term p=0.73, poor adherence: +1.63 mmHg (−2.97 to 6.22), moderate adherence: −1.50 (−7.44 to 4.44), good adherence: +0.74 (−6.14 to 7.62); Activation and ig interaction term p=0.46, poor activation: −0.27 mmHg (−4.04 to 3.50), moderate/ good activation: +2.70 (−3.63 to 9.03). |  |  | RCT | 4/5 |
| Movva et al. (2025) | Mobile app with cataract surgery patients  Mean age: 59.2/58.7  n=360 | 6 weeks |  |  |  | At week 1, 86.7% achieved 6/6 vision (ig=app) vs. 41.1% (cg=standard care), p <0.001) but no significant differences were noted beyond Week 2. It is suggested that early adherence contributed to faster recovery but did not impact long-term vision outcomes. Differences in complication rates (dry eye, cystoid macular edema (CME), posterior capsule opacification (PCO)) were n.s. |  |  | RCT | 2/5 |
| Ni et al. (2018) | Mobile apps with coronary heart disease patients  Mean age: NA  n=50 | 1 month |  |  |  | Comparison of mean changes: SBP (mm Hg) ig change in 15 days -1.33 SD(18.51), in 30 days +0.93 SD(10.40), cg change in 15 days -0.83 SD(19.93), in 30 days -3.76 SD(25.72), t-tests: 15 days p=0.94, 30 days p=0.51 (ns); DBP (mm Hg) ig change in 15 days +7.50 SD(17.43), in 30 days +0.81 SD(10.38), cg change in 15 days +4.50 SD(16.78), in 30 days +8.71 SD(21.43), t-tests: 15 days p=0.62, 30 days p=0.19 (ns); heart rate (bpm) ig change in 15 days +4.92 SD(13.42), in 30 days +4.64 SD(11.89), cg change in 15 days -6.12 SD(12.14), in 30 days -8.06 SD(13.66), t-tests: 15 days p=0.03, 30 days p=0.01, (s.), but decreased in the control group and increased in the intervention group which was significantly lower at the baseline.  Mixed effects regression model:  Difference in the (daily) rate of change between ig and cg (group and time interaction) in SBP +0.17, p=0.46; DBP -0.27, p=0.17; heart rate +0.39 p=0.02, DBP favoring intervention. |  |  | RCT | 2/5 |
| Ni et al. (2022) | Mobile apps with coronary heart disease patients  Mean age: ig 61, cg 62 years  n=230 | 3 months |  |  |  | Comparison of mean changes: SBP (mm Hg) ig change in 60 days -2.14 SD(16.20), in 90 days -2.87 SD(15.10), cg change in 60 days +2.72 SD(16.07), in 90 days +4.38 SD(14.89), t-tests: 60 days p=0.06, 90 days p=0.002 (s.); DBP (mm Hg) ig change in 60 days -2.46 SD(12.49), in 90 days -1.57 SD(12.21), cg change in 60 days +1.87 SD(14.06), in 90 days +2.92 SD(13.99), t-tests: 60 days p=0.04, 90 days p=0.03 (s.); heart rate (bpm) ig change in 60 days -1.46 SD(12.68), in 90 days -1.88 SD(12.88), cg change in 60 days -1.95 SD(9.03), in 90 days -1.32 SD(8.98), t-tests: 60 days p=0.78, 90 days p=0.75, (ns).  Mixed effects regression model:  Difference in the (daily) rate of change between ig and cg (group and time interaction) in SBP -0.08, p<0.001; DBP -0.05, p=0.004; heart rate -0.01 p=0.74, all favoring intervention.  A generalized mixed effect model with a logit link for dichotomized binary normal health outcomes:  Difference between the two groups in the (daily) change of the proportional rate of participants for normal SBP +0.01 p=0.02, DBP +0.006 p=0.32, heart rate +0.003 p=0.60, all favoring intervention. |  |  | RCT | 2/5 |
| Ong et al. (2021) | Mobile apps with high-risk CKD patients (comparison of two apps)  Mean age: eKidneyCare 56, MyMedrec 58 years  n=182 | 12 months |  |  |  | BP (mmHg) Clinic: eKidneyCare 6 mo SBP -0.16 SD(20.1), DBP -0.26 SD(11.1), 12 mo SBP -1.81 SD(22.2), DBP -1.52 SD(12.9) vs. MyMedRec 6 mo SBP -1.21 SD(18.9), DBP -1.78 SD(9.9), 12 mo SBP -2.61 SD(19.4), DBP -1.49 SD(10.3), p-values: 0.78, 0.24, 0.78, 0.91;  Home BP measures:  eKidneyCare 12 mo SBP -2.22 SD(16.9) DBP -0.26 SD(8.9) vs. MyMedRec +0.96 SD(14.1), +0.10 SD(7.1), p-values: 0.33, 0.92.  Laboratory measures: eKidneyCare  hemoglobin (g/dl) +0.01 SD(1.29), potassium (mEq/L) -0.1 SD(0.6), phosphate (mg/dl) +0.03 SD(1.36) vs. MyMedRec +0.25 SD(1.11), -0.1 SD(0.7), -0.19 SD(1.49), p-values: 0.31, 0.71, 0.42. Patient reported: eKidneyCare HADS -0.1 SD(4.6), VR12-PCS -0.1 SD(9.8), VR12-MCS -1.1 SD(8,9), EQ-5D +0.8 SD(0.1) vs. MyMedRec +0.5 SD(5.8), +0.2 SD(7.6), -0.7 SD(10.3), +0.8 SD(0.2), p-values: 0.33, 0.88, 0.46, 0.41. (all ns) |  |  | RCT | 2/5 |
| Ravari et al. (2020) | SMS with hypertension patients  Mean age: ig 63.96, cg 65.48 years  n=100 | 3 months |  |  |  | DBP (s. at 1 month, p=0.0001), SBP (s. at 2 and 3 months, p=0.0001) favored the intervention. |  |  | RCT | 3/5 |
| Santo et al. (2019) | Mobile app with coronary heart disease patients  Mean age: ig 58.4, cg 56.8  n=166 | 3 months |  |  |  | Mean difference (95%CI): SBP −0.89 (−6.97 to 5.20), DBP −0.87 (−4.07 to 2.30), BP< 140/90 mmHg, N/total (%) +1.04 (0.86 to 1.27), Total cholesterol +0.00 (−0.31 to 0.30), LDL-C −0.05 (−0.31 to 0.21), LDL-C <2mmol/L, N/total (%) +0.90 (0.73 to 1.10). (all ns) |  |  | RCT | 4/5 |
| Sikorskii et al. (2018) | Automated reminder calls to cancer patients initiating new oral oncolytic agents  Mean age: 61 years  n=272 | 12 weeks |  |  |  | Symptom severity index: Week 4: 95%CI(-6.02 to 1.83), p=0.21. Week 8: (-9.53 to -1.38), p<0.01. Week 12: (-6.75 to 1.40), p=0.22.  Number of symptoms (above threshold): Week 4: (-0.97 to 0.21), p=0.21. Week 8: (-1.41 to -0.19), p=0.01. Week 12: (-1.02 to 0.21), p=0.19.  (All favoring intervention, but only week 8 results statistically significant.) |  |  | RCT | 4/5 |
| Solmaz & Altay (2024) | Medication reminder wristwatches combined with training for hypertension patients vs. training only vs. no intervention  Mean age: 71.4/72.2 /73.6 years  n=90 | 12 weeks |  |  |  | Wristwatches improved SBP (from 148.7 (SD 9.4) to 120.0 (SD 9.5)) and DBP (from 93.0 (SD 7.5) to 67.7 (SD 7.7)) in pre-post testing (both s., p<0.001) whereas the training only group which lacked the reminder wristwatch, improved also SBP (from 146.3 (SD 9.6) to 126.0 (SD 13.0)) and DBP (from 92.0 (SD 4.8) to 76.7 (SD 10.3 (both s., p<0.001).  Comparable differences are favoring reminder wristwatches over training only (but snr).  The control group with neither training nor reminder wristwatches improved also SBP (from 146.7 (SD 8.0) to 142.7 (SD 11.1)), p=0.083, and DBP (from 94.7 (SD 5.1) to 81.7 (SD 12.6)), p<0.001.  Tests for the differing of SBP and DBP post-test means in the tree group were s., p<0.001, but did not separately test medication reminder wristwatch group (with training) compared to training. |  |  | RCT | 3/5 |
| van de Hei (2025) | Reminders for asthma patients. Ig: Electronic monitoring device and mobile application including visualization of inhaler use, reminders, missed dose and overuse messages, motivational nudge messages, and possibility to track symptoms and triggers. Cg: Inhaler use passively monitored, connected to a smartphone application in which actuation data were not visible.  Mean age: 47.5 years  n=164 | 12 months | Estima-ted cost per 0.5 point de-crease in ACQ-5 (the MCID) was €278. |  |  | Asthma control (ACQ-5): Significantly better overall ig vs cg across 12 months (estimated marginal means (EMM): 1.31 95%CI, 1.18-1.44) vs 1.56 95%CI, 1.44-1.68; p=0.0056); group×time interaction (p=0.251) not included. At 12 months, 42.9% vs 20.0% achieved an improvement ≥ minimal clinically important difference (MCID 0.5); OR=3.00 (95% CI 1.13–8.35).  In addition, 15.2% and 4.9% of the intervention and control group participants, respectively, shifted from being uncontrolled (ACQ-5 ≥ 0.75) to controlled (ACQ-5 <0.75) (OR=3.48; 95%CI=0.63-19.26).  Asthma related quality of life (Mini AQLQ): Mean difference narrowly non-significant EMM intervention group 5.69, 95% CI, 5.55-5.82 vs control group 5.51, 95% CI 5.39-5.63) (p=0.0530). However, significantly more patients reached the MCID (≥0.5): ig 44.8% vs cg 22.9%; OR = 2.73 (95% CI 1.02–7.54) at 12 months (n=77).  Exacerbations:  self-reported: patients ig: 7/35 vs. cg: 8/42, total exacerbations: ig: 12 vs. cg: 12 (p=0.8448);  from general practitioner electronic health records: patients: ig: 2/32 vs. cg: 5/41, total exacerbations: ig: 6 vs. cg: 6 (p=0.8682). No serious adverse events reported.  Linear mixed model subgroup analyses found no modification of the intervention effect on ACQ 5 by attitude, self-efficacy, or medication beliefs (all interaction p>0.05). Illness perception and eHealth literacy modification of the intervention effect on ACQ-5 were plotted also but reported to have no significance testing.  Work productivity and activity impairment domains (absenteeism, presenteeism, overall and activity impairment): no statistically significant between group differences at any timepoint: baseline, three, six, nine and 12 months (T0–T12). 12-month median changes were equal for absenteeism, presenteeism and activity impairment, while the overall impairment median change (n.s.) slightly favored control. Participants with productivity loss: no significant between group differences (T0–T12). From T0 to T12, absenteeism cases decreased from 10 to 3 in cg and 8 to 5 in ig (larger reduction in cg), presenteeism cases decreased from 28 to 25 in cg and 27 to 18 in ig(larger reduction in ig). Median loss (change) among affected participants from T0 to T12: absenteeism favors control (75.7 to 28.6 (cg) vs 50 to 46.7 (ig)), presenteeism favors intervention (20.0 to 20.0 (ig) vs 30 to 40.0 (cg)), snr. |  |  | RCT | 2/5 |

**Appendix Table A4: Reminder systematic reviews**

| Reminder Review | Intervention  details | N: included/  criteria/  (s.) | Cost | Outcomes  Service use | & Primary  Patient satisfaction and carer burden | Findings  Health | Prof. well-being | Equity | AMSTAR2 Quality |
| --- | --- | --- | --- | --- | --- | --- | --- | --- | --- |
| Adler et al. (2017)/Redfern et al. (2024) (update of Adler et al.) | SMS-intervention with heart disease patients  Reported sample size, intervention length and mean age: n=200, 2 mo, 56/57.6 years, Kamal et al. (2015);  n=62, 8 w, 56/59 years, Khonsari et al. (2015)  n=230, 3 mo, 61/62 years, Ni et al. (2022) | 18/3/2 |  | Khonsari et al. (2015): hospital re-admission rate, effect favoring SMS group but ns. (not in review) |  | Kamal et al. (2015): mean difference of diastolic blood pressure (n.s.) favoring intervention: 2.6 mmHg (95% CI; −5.5 to 0.15)  Khonsari et al. (2015): deaths due to ACS complication (0 vs. 2) favoring intervention; NYHA classification of symptoms (result not found in review): heart functional status (s.) favoring the SMS intervention  Ni et al. (2022): heart rate (n.s), blood pressure (s.), but results not in the Redfern et al. (2024) review. Ni et al. (2022) evaluated as original study. |  |  | 13/16 |
| Al-Arkee et al. (2021)  Including 8 original studies: Brath et al. (2013), Chandler et al. (2019), Frias et al. (2017), Liu et al. (2019),  Morawski et al. (2018), Ni et al. (2018), Santo et al. (2019), Sarfo et al. (2019) | Mobile app with cardiovascular disease patients  Reported sample size, intervention length and mean age:  n=77, 20 w, 69.4 years, Brath et al. (2013); n=54, 9 mo, 46.5 years, Chandler et al. (2019); n=118, 12 w, 58.7 years, Frias et al. (2017); n=57, 12 w, 59 years, Liu et al. (2019); n=412, 12 w, 52 years, Morawski et al. (2018); n=50, 30 d, NA, Ni et al. (2018); n=166, 3 mo, 57.9 years, Santo et al. (2019); n=60, 3 mo, 55 years, Sarfo et al. (2019). | 16/8/4 |  |  |  | SBP: Brath Chandler, Frias, Ni, Santo, Sarfo, Morawski  DBP: Brath Chandler, Frias, Ni, Santo, Sarfo (for BP: Brath Chandler, Frias are s.)  TC: Brath (s.), Frias (s.), Liu (s.), Santo (ns.)  LDL-C: Frias (s.)  TG: Liu (ns.)  Meta-analyses: (all ns.)  (Liu + Santo favoring intervention) LDL-C: -0.19 CI(-0.49 to 0.12) and TC: -0.38 CI(-1.14 to 0.38)  (Morawski + Santo + Sarfo favoring intervention) SBP: -0.74 (-3.49 to 2.01)  (Santo + Sarfo favoring control) DBP: +0.46 (-3.90 to 4.82)  Individual results:  SBP: Santo -1.00 (-7.03 to 5.03), Sarfo -4.70 (-16.89 to 7.49), Morawski -0.40 (-3.59 to 2.79)/ -0.50 (-3.54 to 2.54), Frias -5.70 (-17.04 to 5.64) (all ns. and favoring intervention), Chandler et al. (2019) favors intervention,  Brath et al. (2013) crossover design shows a decrease at intervention end.  DBP: Santo et al. (2019) -0.90 (-4.08 to 2.28), Sarfo et al. (2019) +4.10 (-3.24 to 11.44), Frias et al. (2017) -2.80 (-10.43 to 4.83) (all ns.), Chandler et al. (2019) favors intervention, Brath et al. (2013) crossover design shows a decrease at intervention end.  LDL-C: Liu et al. (2019) -0.38 (-0.76 to 0.00), Santo et al. (2019) -0.06 (-0.32 to 0.20), Frias et al. (2017) -0.30 (-1.01 to 0.41) (all ns., favoring intervention)  Brath et al. (2013) (crossover design) decreased to intervention end.  HDL-C: Brath et al. (2013) (crossover design) increased to intervention end.  Total cholesterol: Santo et al. (2019) +0.00 (-0.30 to 0.30) (ns.), Liu et al. (2019) -0.78 (-1.17 to -0.39) (s.), Frias et al. (2017) favors intervention, cross-over design Brath et al. (2013) shows decrease from baseline to intervention end.  TG: Liu et al. (2019) favors intervention.  HbA1c, %: Frias et al. (2017) favors intervention, Brath et al. (2013) decreased to intervention end.  Morawski et al. (2018), Santo et al. (2019), Ni et al. (2018) also evaluated as original studies.  Morawski et al. (2018) and Frias et al. (2017) also included in Yap et al. (2022) systematic review. |  |  | 5/16 |
| Aung et al. (2024) | Remotely monitored digital inhaler and mobile app: Control (Criner cg, Yawn pretest) had similar devices but without medication reminder functionality)  Reported sample size, intervention length and mean age:  n=138, 6 months follow-up, mean age: ig 66.6, cg 66.7 years, Criner et al. (2021);  n=122 (before-after study, no control group), follow-up 6 months (follow-up from 12 to study exit (maximum 24 weeks)), mean age: 65.2 years, Yawn et al. (2021) |  |  |  |  | Criner: Mean±SD CCQ scores: ig 2.18±0.82 versus 2.39±1.17, p=0.80 (n.s., favoring control)  Yawn: Mean±SD COPD Assessment Test (CAT) score: pre 21±7.4 versus post 20.2±8.3. ns. |  |  | 8½/16 |
| Choi et al. (2020) | Mobile app with hypertension and ischemic heart disease patients  Reported sample size, intervention length and mean age: n=690, 3 mo, 57.9/56.9 years, Albini et al. (2016); n=42, 6 mo, 56 years, Anglada-Martinez et al. (2016); n=38, 6 mo, 47.5/48.5 years, Davidson et al. (2015); n=50, 10 mo (activation phase 3 mo), 53 years, Patel et al. (2013); | 17/4/3 |  | Note: Health care resource use results in Anglada-Martinez et al. (2016) but reported p-values appear erroneous (not found in included reviews) |  | Davidson et al. (2015): BP control (s.):  70.6% ig vs. 15.8% cg (<140/90 mmHg) at 1 mo (p<0.001), 94.4% ig vs. 41.2% cg at 6 mo (p<0.003)  Albini et al. (2016):  Office BP control (s.): 40.0% cg vs. 72.3% ig at 6 mo follow-up (p<0.0001) - At same time Home BP control ig: 87.5%  Patel et al. (2013): sBP (s., decreasing, compared with baseline), dBP (n.s.)  Anglada-Martinez et al. (2016): sBP (ns.), dBP (ns.) in hypertension/dyslipidemia, cholesterol and TG in dyslipidemia (ns.), QoL (ns.)  Anglada-Martinez et al. (2016) and Patel et al. (2013) are also included in Ng et al. (2020) review. Davidson et al. (2015) also included in Yap et al. (2022) systematic review. |  |  | 6/16 |
| Cross et al. (2020)  Including only  Chrischilles et al. (2014) | Personal health record with medication safety messages  Reported sample size, intervention length and mean age:  n=1163, 6 mo, 72.5/ 72 years, Chrischilles et al. (2014) | 50/1/0 |  |  |  | Chrischilles et al. (2014): self-reported side-effects: personal health records 100/802  (12.9%) vs usual care  33/273 (12.2%) (snr) |  |  | 14/16 |
| Ng et al. (2020) | Mobile apps  Reported sample size, intervention length and mean age:  n=42, 6 mo (ig had Medplan 3 mo after cg usual care), 56 years, Anglada-Martinez et al. (2016); n=32, 90 d, 56 years, Feng et al. (2017); n=28, 12 w (ig daily monitored by AI platform), 57 years, Labovitz et al. (2017); n=102, 3 mo, 70.9/72.9 years, Mira et al. (2014); n=50, 10 mo, 53 years, Patel et al. (2013). | 21/5/3 |  |  |  | Mira et al. (2014): Pre–post difference: HbA1c IG: −0.4 and CG: 0.3, SBP IG: 2.3 and CG: 3.2, DBP IG: 1.7 and CG: 0.8, self-perceived health status IG: 3.3 and CG: 0.9. (All ns.),  IG cholesterol level improved by 5%. Pre–post difference: cholesterol IG: 5.7 and CG: −3.5 (p=0.04).  Patel et al. (2013):  Baseline systolic BP 144/89 significantly higher than IG1 (3 months using reminder application) 136/84 (p=0.04), IG2 (3 months after removal of the application) 135/85 (p=0.01), and CG 3 months prior study 137/85 (p=0.31).  Feng et al. (2017): Endoscopic findings: ns. except granulation score in CG increased more than IG (p<0.001), SNOT-20 score (ns.)  Anglada-Martinez et al. (2016): SBP IG: 131.3 and CG: 130.2, DBP IG: 75.4 and CG: 79.9, Cholesterol IG: 147.2 and CG: 207, Triglyceride IG: 185 and CG: 263.5, (all ns.). EQ-5D questionnaire (ns), viral load IG: <37 and CG: <37 (snr)  Labovitz et al. (2017): Activated partial thromboplastin time IG: 41.7 and CG: 48.4 (snr). Prothrombin time IG: 35.1 and CG: 32.9 (snr) |  |  | 6½/16 |
| Stevenson et al. (2019) | Text message reminder: Jammalamadaka et al. (2015);  Electronic monitoring device, SIM-pill system with reminders vs. cg without reminders: Potter et al. (2016);  Wireless pill bottle with reminders vs. cg without reminders: Reese et al. (2017);  Mobile app: Ong et al. (2017)  Reported sample size, intervention length and mean age: n=40, 7 d, 48/62 years, Jammalamadaka et al. (2015); n=89, 3 years, NA, Potter et al. (2016); n=120, 6 mo, 50/49 years, Reese et al. (2017); n=182, 6 mo, NA, Ong et al. (2017) | 43/4/0 |  |  |  | Jammalamadaka et al. (2015): Serum phosphate (Intervention 6.00 (SD=1.2) - Control 6.19 (SD=0.76), p=0.76, n.s.)  Potter et al. (2016): Number of rejection episodes: ig 0/20 – cg without reminders 9/26, p=na, CI(na).  Reese et al. (2017): Coefficient of variation of TAC blood concentrations (no results found)  Ong et al. (2017): SBP and DBP (s.), median reductions: SBP: ig app with feedback vs cg more passive app -5 mmHg, p=0.05; for DBP: -3.5 mmHg, p=0.03. |  |  | 12/16 |
| Yap et al. (2022) | Mobile app/SMS, hypertension patients.  Reported sample size, intervention length and mean age:  n=123, 1 mo, 49 years, Buis et al. (2017); n=38, 6 mo, 47.5/48.5 years, Davidson et al. (2015); n=118, 3 mo, 57.8/61.6 years, Frias et al. (2017); n=41, 3 mo, 51.7/52.4 years, Morawski et al. (2018) | 74/4/1 |  |  |  | Buis et al. (2017): SBP mean (SD) ig mmHg 140.2 (21.6) vs 140.4 (21.6), mean difference: -0.20, 95%CI(-8.28 to 7.88), n.s., favoring intervention;  DBP mean (SD) ig mmHg 90.2 (13.6) vs 90.4 (11.8), mean difference: -0.20, 95%CI(-4.95 to 4.55), favoring intervention (n.s.)  Morawski et al. (2018): SBP mean (SD) ig mmHg 140.8 (15.7) vs 141.2 (17.3), mean difference: -0.40, 95%CI(-3.60 to 2.80), n.s., favoring intervention; BP control, non-event: ig 67/209 vs 69/202, OR=1.10, 95%CI(0.73 to 1.66), favoring standard care (n.s.)  Frias et al. (2017): BP control, non-event: ig 65/80 vs 9/27, OR=0.12, 95%CI(0.04 to 0.31), favoring intervention (s.)    Buis et al. (2017) and Morawski et al. (2018) evaluated also as individual studies. Frias et al. (2017) and Morawski et al. (2018) are also included in Al-Arkee al. (2021) systematic review. This review Yap et al. (2022) reported no results for Davidson et al. (2015) but it is included also in Choi et al. (2020) systematic review. |  |  | 9/16 |

N: included/criteria/(s.) =For a systematic review: number of studies/ number of studies that meet inclusion criteria/ number of studies with criteria meeting statistically significant results, SBP=systolic blood pressure, DBP=diastolic blood pressure, TC=total cholesterol, LDL-C=low-density lipoprotein cholesterol, HDL-C=high-density Lipoprotein cholesterol, HbA1c=glycated hemoglobin, TG=triglyceride, snr=significance not reported, na=not available, SD=standard deviation, CI=confidence interval, SE=standard error, mo=months, d=days, w=weeks, ig=intervention group, cg=control group

**Search strategy:**

The search terms were: (effect* OR cost* OR financ* OR pric* OR adher*) AND (remind* OR dispens*) AND (sms OR smartphone OR tablet* OR automat* OR robot* OR tech* OR electr* OR device OR digital OR application* OR manag*) AND (medic* OR drug* OR pharma*) AND (home OR "assisted living" OR "independent living" OR ehealth OR mhealth OR "informal care*" OR nonhospital OR "self-manage*") AND (aging OR ageing OR senior* OR elderly OR polypharmacy OR multimorbidity) except for Scopus for which the first AND was replaced by W/1000 and all the other AND's by W/100.

For the Cochrane database we additionally filtered out protocols, editorials, special collections and clinical answers. The searches were conducted for the period 1.1.2017-29.9.2025 and no language restrictions were made. Note that results on older original articles were accounted for as part of included systematic reviews published during that period.

**Additional references:**

Chrischilles EA, Hourcade JP, Doucette W, Eichmann D, Gryzlak B, Lorentzen R, et al. Personal health records: a randomized trial of effects on elder medication safety. Journal of the American Medical Informatics Association 2014;21(4):679-86

Jammalamadaka D, Nahman NS, White JJ. "Binder Reminders" for persistent hyperphosphatemia in hemodialysis patients: a fellow's quality improvement product [abstract no: PUB514]. Journal of the American Society of Nephrology 2015;26(Abstracts):1007A. [CENTRAL: CN-01658469]

Labovitz DL, Shafner L, Reyes Gil M, Virmani D, Hanina A. Using artificial intelligence to reduce the risk of nonadherence in patients on anticoagulation therapy. Stroke. 2017;48(5):1416–1419

Reese PP, Bloom RD, Trofe-Clark J, Mussell A, Leidy D, Levsky S, et al. Automated reminders and physician notification to promote immunosuppression adherence among kidney transplant recipients: a randomized trial. American Journal of Kidney Diseases 2017;69(3):400-9.
